# A telomere-to-telomere reference genome provides genetic insight into the pentacyclic triterpenoid biosynthesis in *Chaenomeles speciosa*

**DOI:** 10.1093/hr/uhad183

**Published:** 2023-09-14

**Authors:** Shaofang He, Duanyang Weng, Yipeng Zhang, Qiusheng Kong, Keyue Wang, Naliang Jing, Fengfeng Li, Yuebin Ge, Hui Xiong, Lei Wu, De-Yu Xie, Shengqiu Feng, Xiaqing Yu, Xuekui Wang, Shaohua Shu, Zhinan Mei

**Affiliations:** College of Plant Science & Technology, Huazhong Agricultural University, Wuhan 430070, China; Wuhan Carboncode Biotechnologies Co., Ltd., Wuhan 430070, China; Sinopharm Zhonglian Pharmaceutical Co., Ltd., Wuhan 430070, China; College of Plant Science & Technology, Huazhong Agricultural University, Wuhan 430070, China; College of Horticulture & Forestry, Huazhong Agricultural University, Wuhan 430070, China; College of Plant Science & Technology, Huazhong Agricultural University, Wuhan 430070, China; College of Plant Science & Technology, Huazhong Agricultural University, Wuhan 430070, China; College of Plant Science & Technology, Huazhong Agricultural University, Wuhan 430070, China; School of Pharmaceutical Science, South-Central Minzu University, Wuhan 430074, China; School of Pharmaceutical Science, South-Central Minzu University, Wuhan 430074, China; Wuhan Carboncode Biotechnologies Co., Ltd., Wuhan 430070, China; Department of Plant and Microbial Biology, North Carolina State University, Raleigh, NC 27695, USA; College of Plant Science & Technology, Huazhong Agricultural University, Wuhan 430070, China; College of Horticulture, Nanjing Agricultural University, Nanjing 210095, China; College of Plant Science & Technology, Huazhong Agricultural University, Wuhan 430070, China; College of Plant Science & Technology, Huazhong Agricultural University, Wuhan 430070, China; College of Plant Science & Technology, Huazhong Agricultural University, Wuhan 430070, China

## Abstract

*Chaenomeles speciosa* (2*n* = 34), a medicinal and edible plant in the Rosaceae, is commonly used in traditional Chinese medicine. To date, the lack of genomic sequence and genetic studies has impeded efforts to improve its medicinal value. Herein, we report the use of an integrative approach involving PacBio HiFi (third-generation) sequencing and Hi-C scaffolding to assemble a high-quality telomere-to-telomere genome of *C. speciosa.* The genome comprised 650.4 Mb with a contig N50 of 35.5 Mb. Of these, 632.3 Mb were anchored to 17 pseudo-chromosomes, in which 12, 4, and 1 pseudo-chromosomes were represented by a single contig, two contigs, and four contigs, respectively. Eleven pseudo-chromosomes had telomere repeats at both ends, and four had telomere repeats at a single end. Repetitive sequences accounted for 49.5% of the genome, while a total of 45 515 protein-coding genes have been annotated. The genome size of *C. speciosa* was relatively similar to that of *Malus domestica*. Expanded or contracted gene families were identified and investigated for their association with different plant metabolisms or biological processes. In particular, functional annotation characterized gene families that were associated with the biosynthetic pathway of oleanolic and ursolic acids, two abundant pentacyclic triterpenoids in the fruits of *C. speciosa*. Taken together, this telomere-to-telomere and chromosome-level genome of *C. speciosa* not only provides a valuable resource to enhance understanding of the biosynthesis of medicinal compounds in tissues, but also promotes understanding of the evolution of the Rosaceae.

## Introduction

*Chaenomeles speciosa* (Sweet) Nakai, known as Mugua or Fructus Chaenomelis, is a deciduous shrub in the Rosaceae. It is a diploid species (2*n* = 34), which is native to China and cultivated worldwide. The *Chaenomeles* genus includes five species: *C. thibetica* Yü, *C. japonica* (Thunb.) Lindl. ex Spach, *C. cathayensis* (Hemsl.) Schneider, *C. speciosa* (Sweet) Nakai, and *C. sinensis* (Thouin) Koehne. Of these, *C. speciosa* is an edible medicinal plant. Its fruits have a sour flavor and are one of several important parts of this plant that have been used in traditional Chinese medicine (TCM) for over a thousand years [1]. Its dry fruits are noted to be able to calm the liver, relax the muscles and tendons, harmonize the stomach, and eliminate dampness. The medicinal applications of its dry fruits in TCM include the treatment of asthma, hepatitis, dyspepsia, dysentery, enteritis, and rheumatoid arthritis [2]. In addition, its fruits are widely used in jams and jellies.

To date, the medicinal uses of dry fruits have prompted multiple studies for understanding their active compounds. Phytochemical analyses have reported that the fruits of *C. speciosa* contain triterpenoids, phenolic and phenylpropionic acids, flavonoids, saccharides, and alkaloids [1, 3–5]. Of the triterpenoids, the Chinese Pharmacopoeia has specified that oleanolic and ursolic acids are two characteristic metabolite markers of *C. speciosa* [2, 6]. Recent metabolomics analyses further revealed that its fruits were rich in health-beneficial metabolites with medicinal uses [7], such as anti-inflammatory, anti-nociceptive, antimicrobial, antiviral, antioxidant, anti-influenza, and immunoregulatory activities [2, 3, 8, 9]. Thesefindings indicate the significance of further investigations in understanding the phytochemical basis of the medicinal uses of *C. speciosa*. Given that the current understanding of plant metabolism in this medicinal plant is limited, investigations, such as genomics, elucidation of the biosynthesis of active phytochemicals, metabolic engineering, and genetics, are necessary for the successful production of active compounds.

The Rosaceae is a large family of over 3000 species, of which apple, peach, pear, plum, strawberry, cherry, raspberry, and rose are common economic crops benefiting human health. Accordingly, the Rosaceae is appropriate for investigations of fruit diversity, domestication, and evolution [10]. To date, the genomes of several species have been sequenced and showed value in enhancing the understanding of genetics for breeding and other research efforts [10]. Based on short reads generated by next-generation sequencing technologies, those sequencing investigations have provided genome annotation pipelines for related plants [10]. In addition, despite certain technological limitations, those complete, accurate, and contiguous representative genome sequences provide valuable references for further genomic analysis of other species [11]. More importantly, those available genome sequences are useful references for genome annotation of *Chaenomeles* species, none of which has been sequenced, to our knowledge.

To date, advances have been made in long-read sequencing technologies and assembly pipelines. These new technologies, such as high-fidelity (HiFi) sequencing, a third-generation approach, have allowed the generation of genomic sequences for several chromosome-scale genome assemblies with increasing completeness, contiguity, and accuracy [12–14]. More importantly, HiFi sequencing has been used to develop telomere-to-telomere (T2T) and gap-free reference genomes of crops and has shown the ability to identify unique genes and structural variations in repetitive regions [15, 16]. Thus, we report the use of this new technology to sequence and assemble the genome of *C. speciosa*, a heterozygous diploid. The resulting T2T genome was of high quality and provided useful information to understand the biosynthesis of triterpenoids in *C. speciosa*. In addition, these genomic sequences provided valuable resources to understand the effects of domestication on sequence variations and plant evolution in the Rosaceae.

## Results

### *De novo* assembly of the *C. speciosa* genome

**Figure 1:**
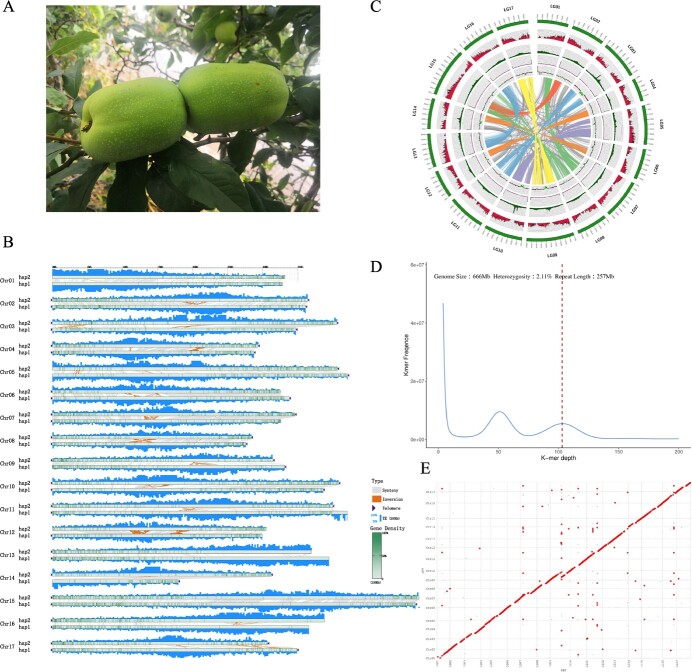
Genome survey and assembly of *Chaenomeles speciosa.* (**A**) Greenish fruits*,* (**B**) collinearity of the two sets of *C. speciosa* haploid chromosomes. The collinearity is based on telomere, repeat sequence distribution, and gene density between the two sets of chromosomes. (**C**) High-quality genome assembly of *C. speciosa*. The genome diagram features five circles. The outmost greenish circle comprises 17 chromosomes. The remaining circles represent distributions of genes, transposons, telomere repeats, and GC content along with the chromosomes. The center represents the synteny blocks among the chromosomes. (**D**) Plot showing heterozygosity resulting from *k*-mer analysis. (**E**) Plot showing the similarity of syntenies between the genomes of apple and *C. speciosa.*

A combination of short reads, HiFi, and Hi-C sequencings was used to sequence and assemble the genome of *C. speciosa* (Fig. 1A). First, we completed short-read sequencing and obtained paired-end reads of 660 Gb. The K-mer survey results (*k* = 21) showed that the genome size was ~650.40 Mb with a heterozygosity rate of 2.1% and we identified a heterozygous peak and a homozygous peak at a depth of ~50 and 100, respectively (Fig. 1B and D). Detailed analysis revealed that the genome contained ~257.0 Mb repeated sequences. Next, we used high-quality DNA samples to construct a SMRTbell library and sequenced it with a PacBio Sequel II system. This sequencing generated a total of 18.8 Gb HiFi reads with an N50 length of 19.0 kb (28.92× coverage). We then used the pipeline of Hifiasm to assemble the HiFi reads and obtained a primary assembly containing 167 contigs with a cumulative size of 650.4 Mb (Table 1). The longest contig was 51.8 Mb in length, with 50% of contigs being more than 35.5 Mb long, and 19 covering 90% of the total length (Table 1).

**Table 1:** Data summary for two haplotype assemblies

	Hap1			Hap2		
	Contigs	Scaffolds	Pseudo-chromosomes	Contigs	Scaffolds	Pseudo-chromosomes
No. of assembly	230	125	17	97	42	17
Total length (bp)	605 875 599	605 928 099	592 846 119	632 365 991	632 393 491	608 284 049
N50 (bp)	25 567 090	36 115 581	36 115 581	29 342 999	36 916 915	36 916 915
L50	10	7	7	8	7	7
N90 (bp)	5 854 963	29 924 437	29 924 437	8 796 568	30 504 571	30 700 526
L90	30	14	14	25	14	15
Gap Number	0	103	61	0	45	26
Max length (bp)	38 639 417	51 774 635	51 774 635	40 832 825	52 302 129	52 302 129
GC content (%)	37.59	37.59	37.41	37.42	37.25	37.25
No. of protein-coding genes	40 874			43 836		
BUSCO (genome)	C: 96.2% (S: 66.4%, D: 29.8%), F: 0.8%, M: 3.0%, n:1440			C: 96.2% (S: 64.3%, D: 31.9%), F: 1.3%, M: 2.5%, n:1440		
BUSCO (protein-coding genes)	C: 90.1% [S: 64.5%, D: 25.6%], F: 5.3%, M: 4.6%, n:1440			C: 91.6% [S: 64.2%, D: 27.4%], F: 4.9%, M: 3.5%, n:1440		

To anchor the contigs, a total of 40.0 Gb paired-end reads were generated from the Hi-C library. After cleaned reads were filtered using HiCUP, the resulting data showed that 94.2% were valid pairs. All valid pairs were used for scaffolding to obtain a total of 122 scaffolds, of which 16 covered 90% of the total length (Table 2). Based on Hi-C sequencing data, 25 scaffolds were anchored onto 17 pseudo-chromosomes (Fig. S1, see online supplementary material). This chromosome-level genome assembly had 632.3 Mb sequences with a scaffold N50 size of 35.8 Mb and a GC content of 37.3% (Table 2). Further sequence analysis characterized that the lengths of 17 pseudo-chromosomes ranged from 24.5 to 51.8 Mb. Of the 17 pseudo-chromosomes, twelve, four, and one comprised a single large contig, two contigs, and four contigs, respectively (Table S1, see online supplementary material). Analysis of those sequences located at the ends of the pseudo-chromosomes showed that 11 chromosomes had telomere repeats in both ends and four chromosomes had telomere repeats in a single end (Fig. 1C; Table S1, see online supplementary material). Two chromosomes, LG01 and LG06, lacked telomere repeats. The Centromics program was used to identify the centromeres [17], with the results demonstrating the presence of centromeres on every chromosome (Fig. S2, Table S2, see online supplementary material). In addition, seven gaps were identified in the assembled 17 chromosomes (Table S3, see online supplementary material). To characterize gap-flanking 20-kb (upstream 10-kb and downstream 10-kb) region sequences, a collinear analysis was carried out using the MUMmer program. The resulting data indicated a large number of dispersed repeats and inverted repeats in these flanking 20-kb region, such as the 11 kb- ~ 20 kb of gap (LG08Gap1::LG08:16430026–16 450 026) on chromosome 8 was a reverse repeat, and this region also has a copy (dispersed repeat) at the position of the 0 kb–9 kb of the third Gap (LG09Gap3::LG09:26623338–26 643 338) on chromosome 9 (Fig. S3, see online supplementary material). Furthermore, the analysis of GC revealed that the GC content ranged from 35.16% to 39.92% in the gap-flanking sequences, with an average value of 37.54%. The GC content in the 17 chromosomes ranged from 36.29% to 37.94%, with an average value of 37.31%. Student’s *t*-test analysis did not indicate any significant difference in the GC content between gap-flanking sequences and whole chromosomes (*P* = 0.7391). In addition, we extracted 10 kb-long nucleotides in seven gap-flanking sequences and obtained eight genes, which were annotated to be one transcription factor, receptors, and others (Table S4, see online supplementary material).

**Table 2:** Data summary for genome assembly of *Chaenomeles speciosa*

	*C. speciosa* genome		
	Contigs	Scaffolds	Pseudo-chromosomes
No. of assembly	167	122	17
Total length (bp)	650 391 965	650 410 993	632 305 965
N50 (bp)	35 454 787	35 780 769	35 780 769
L50	8	8	8
N90 (bp)	16 814 540	29 516 134	32 355 895
L90	19	16	15
Gap number	0	45	7
Max length (bp)	51 822 334	51 822 334	51 822 334
GC content (%)	37.39	37.47	37.31
No. of protein-coding genes	45 515		
BUSCO (genome)	C: 96.5% (S: 65.0%, D: 31.5%), F: 1.0%, M: 2.5%		
BUSCO (protein-coding genes)	C: 93.7% [S: 62.0%, D: 31.7%], F: 4.1%, M: 2.2%		

Next, Illumina sequencing data were generated to build a Hi-C library. HiFiasm in combination with HiFi data provided precise local haplotype information, and Hi-C data provided long-range interaction information to achieve global phasing of the genome, which identified two haplotypes of the *C. speciosa* genome, Hap 1 and Hap 2. These comprised 34 chromosomes. The genome size of Hap1 was 592.84 Mb, characterized by 230 contigs (N50 of 25.57 Mb), 125 scaffolds (N50 of 36.12 Mb), and a 37.41% GC content. The genome size of Hap2 was 608.28 Mb, characterized by 97 contigs (N50 of 29.34 Mb), 42 scaffolds (N50 of 36.92 Mb), and a 37.25% GC content (Table 1; Tables S5 andS6, see online supplementary material). Based on these sequences, in Hap1, eight chromosomes had telomere repeats at both ends, and eight chromosomes contained telomere repeats at a single end; in Hap2, 10 chromosomes had telomere repeats at both ends, and five chromosomes included telomere repeats at a single end (Fig. 1B; Tables S7 and S8, see online supplementary material). Further quality evaluation with benchmarking universal single copy ortholog (BUSCO) analysis showed that Hap1 had 96.2% coverage of the embryophyta_odb 10 gene set, compared with 96.2% for Hap2 (Table 2). Finally, based on the genome of Hap1 and Hap2, we assembled the *C. speciosa* genome comprising all 34 chromosomes and constructed a circle map to visualize its genome (Fig. 1C).

We compared the genomes of Hap1 and Hap2 based on the assemblies described above. The data indicated the similarity of the two haplotypes in terms of genome size and number of genes (Table 2). The use of whole-genome alignment further revealed the large-scale conservative collinearity of the two haplotypes (Fig. 1B). We also analysed single nucleotide polymorphisms (SNPs), insertion–deletions (InDels), and structural variation (SV) between the two haplotypes. The data revealed obvious differences between Hap1 and Hap2, including 6 972 451 SNPs, 987 101 InDels, 73 contractions, 5461 insertions, 4587 deletions, 70 inversions, 125 translocations, and 184 duplications (Fig. 1B; Fig. S4, Tables S9–S11, see online supplementary material). These data provide a substantial source of information to understand the genetic variation between the two haplotypes. The characterization of sequences suggested that 258 544 SNPs and InDels (876 start codons, 6614 stop codons, and 10 597 splicing sites, missense variants, and frameshifts) contribute to the diversity of gene functions in *C. speciosa* (~2.7% cultivars).

Amino acid sequences deduced from the Hap1 and Hap2 genomes were used for clustering analysis (mcl program, parameter -I 2.0 -abc). The resulting data returned 28 555 gene families. Among these, 10 487 resulted from the annotation of 26 966 Hap1 and 27 923 Hap2 genes and were characterized as multi-ortholog gene families. In addition, 11 382 gene families were characterized as single-ortholog gene families, in which 2395 gene families annotated from 2526 single genes were Hap1 specific and 4291 gene families annotated from 4531 single genes were Hap2 specific (Tables S12–S14, see online supplementary material). Moreover, we used Kyoto Encyclopedia of Genes and Genomes (KEGG), Gene Ontology (GO), and Protein Family Analysis and Modeling (PFAM) to perform enrichment analysis to characterize the Hap1- and Hap2-specific genes. KEGG results revealed that Hap1-specific genes were enriched in the biosynthesis of amino acids (ko01230), thyroid hormone signaling pathway (ko04919), and plant–pathogen interaction pathways (ko04626), whereas Hap2-specific genes were enriched in spliceosome (ko03040), betalain biosynthesis (ko00965), and vitamin B6 metabolism (ko00750) (Fisher’s exact test, *P* < 0.05) (Figs S5 andS6, see online supplementary material). GO results showed that Hap1-specific genes were enriched in DNA integration (GO: 0015074), cellular response to stimulus (GO: 0051716), and signal transduction functions (GO: 0007165), whereas Hap2-specific genes were enriched in DNA integration (GO: 0015074), cell cycle (GO: 0007049), and DNA metabolic process function (GO: 0006259) (Fisher’s exact test, *P* < 0.05) (Figs S7–S12, see online supplementary material). PFAM results indicated that Hap1-specific and Hap2-specific genes were both enriched in reverse transcriptase (RNA-dependent DNA polymerase) (RVT_2), integrase core domain (rve), and zinc-binding in reverse transcriptase (zf-RVT) (Figs S13 and S14, see online supplementary material).

### Evaluation of the genome quality

To evaluate the quality of the assembly, we completed a BUSCO analysis. The resulting data showed that 96.5% of BUSCOs were present in the assembly (Table 2). The single copy and duplicated genes accounted for 65.0% and 31.5% of the assembled genome, respectively. These data indicated a highly genome-wide duplication of *C. speciosa*. The BUSCO analysis also revealed only 2.5% missing genes and 1% missing fragments, suggesting that the genome assembly was nearly complete.

The long-terminal repeat (LTR) assembly index (LAI) score, read mapping and pseudo-chromosome comparison are three other important approaches to evaluate the quality of genome assembly [18]. First, LTR prediction revealed a LAI score of 11.23. This high score indicated the high quality of this genome assembly. Meanwhile, the paired-end reads used for the genome survey were aligned with the assembly and the results showed high mapping ratio reached 98.02%. The average coverage depth of the second-generation survey data to the genome was 106.33×; distribution depth maps of the data were developed to visualize the sequence quality of the second-generation sequencing from each chromosome (Fig. S15, see online supplementary material). Finally, we compared our sequences with those obtained from second-generation sequencing and used GATK 4.0 for SNP and InDel analysis. This comparison obtained 4386 (average 258 per chromosome) homozygous SNPs and InDels from 17 chromosomes. In addition, this comparison showed that the proportion of SNPs and InDels in terms of the entire chromosome size was 6.936515e^−06^, which decreased to 5.021475e^−06^ to 8.560801e^−06^, indicating the proportion values of each chromosome. Further statistical analyses showed that the accuracy of the assembled single base of the chromosome was more than 99.999%. (Table S15 and Fig. S16, see online supplementary material). These statistical data indicated the high accuracy of the assembly.

In addition, the pseudo-chromosomes of *C. speciosa* were compared with those of apple to understand any structural and sequence similarities. Two genomes showed a close one-to-one synteny relationship at the chromosome level (Fig. 1E, Table S16). Analysis with MCScanX detected 231 collinearity blocks (Table S17, see online supplementary material), which contained an average of 375 genes. The largest block contained 2670 genes and the smallest contained 33 genes. These blocks covered 26 391 genes of *C. speciosa* (57.98% of total genes) and 25 148 genes of apple (57.98% of total genes). In addition, the protein sequences between apple and *C. speciosa* were compared with Blast (threshold: e-value <1e-5, min_coverage >40%). The resulting data revealed 10 029 gene clusters (34 352 *C. speciosa* genes and 34 991 apple genes), in which 9704 were *C. speciosa*-specific genes, whereas 10 521 were apple-specific genes. To understand their potential functions, we carried out enrichment analysis for *C. speciosa*-specific genes. The results from KEGG showed that enriched *C. speciosa*-specific genes were associated with apoptosis (KO04210), carbon metabolism (ko01200), and gap junction and other metabolic pathways (Ko04540) (Fisher’s exact test, *P* < 0.05). The results from the conserved Pfam domain analysis showed that enriched functions of those *C. speciosa*-specific genes were MULE transposase domains, SWIM zinc finger proteins, and reverse transcriptases (*P* < 0.05) (Table S18, see online supplementary material). The GO function analysis showed that those genes were enriched in RNA–DNA hybrid ribonuclease activity (GO: 0004523), endonuclease activity (GO: 0016893), endoribonuclease activity (GO: 0004521), and others (Tables S18–S21, see online supplementary material) (Fisher’s exact test, *P* < 0.05). Taken together, these results not only indicated the high contiguity, completeness, and accuracy of the assembled *C. speciosa* genome, but also showed that this assembly is a valuable reference genome.

### Annotation of genomic elements

Genomic elements were annotated to characterize the assembly. The repetitive sequences in the assembled genome were identified using both *de novo* prediction and homology-based searches. In total, 312.9 Mb repetitive sequences were identified, accounting for 49.5% of the genome. Sequence analysis revealed that transposable elements (TEs) formed the primary part of the repetitive sequences. RNA retrotransposons (Class I) were the main part of Tes. Of these, LTRs were the most abundant, accounting for 36.3% of the whole genome (Table S22, see online supplementary material). The dominant LTR retrotransposons were *Gypsy* elements (22.7%), followed by *Copia* elements (7.3%). In addition to Class I, DNA transposons (Class II) accounted for 1.0% of the genome, with PIF-Harbinger being the most abundant.

The gene models were predicted using a combination of *ab initio*, homology-based searches, and Iso-seq data. The resulting data predicted 45 515 high-confidence protein-coding genes (representing 19.0% of the assembly) with an average length of 2636 bp in the *C. speciosa* genome. All these coding genes were used for functional annotation via seven public databases. The results showed that 93%, 90%, 70%, 67%, 48%, 43%, and 29% of coding genes were annotated via NCBI, NR database, TrEMBL database, Pfam database, SwissProt protein database, KOG database, GO database, and KEGG database, respectively. All these databases allowed the functional annotation of 93% coding genes. In addition, the gene models obtained 93.7% of BUSCOs, including 62.0% single-copy genes and 31.7% duplicated genes (Table 2).

In addition, the genome assembly identified 4205 ribosomal RNAs (rRNAs), 685 transfer RNAs (tRNAs), 274 small-nuclear RNAs (snRNAs), and 739 small nucleolar RNA (snoRNA). Based on sequence analysis and annotations, we developed a Circos plot (Fig. 1C) that visualized the distributions of genes, transposons, telomere repeats, and GC content along with the chromosomes and the synteny block among the chromosomes. In addition, the data of the synteny block analysis included in the plot characterized the sequence similarities of the genome regions connected with lines. The synteny block analysis further revealed that homologous chromosome pairs, such as LG01 and LG7, LG02 and LG15, LG03 and LG11, LG04 and LG12, LG05 and LG10, LG06 and LG14, LG08 and LG15, LG09 and LG17, LG13 and LG16, were derived from tetraploidization event [19] (Fig. 1C).

### Orthologous clustering and phylogenetic status of *C. speciosa*

**Figure 2:**
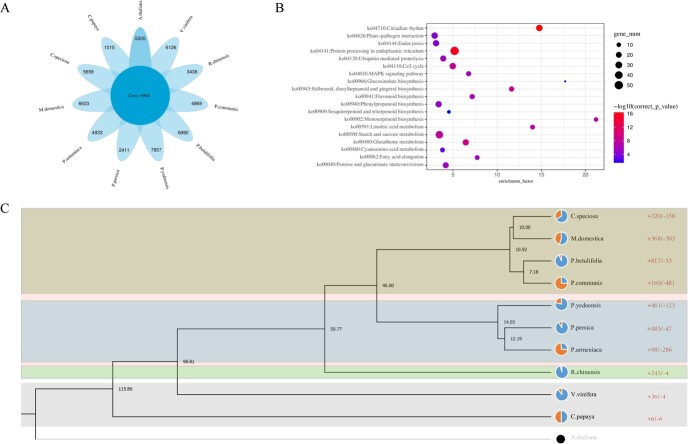
Orthologous clustering and phylogenetic analysis of the *Chaenomeles speciosa* genome. (**A**) The flower-shaped diagram shows the core orthogroup (in the center) shared by *C. speciosa* and 10 other plant species and 11 petal parts formed by 11 species-specific orthogroups. (**B**) Enrichment analysis via KEGG characterizes that expanded gene families in the *C. speciosa* genome are associated with 17 different functions. (**C**) A phylogenetic tree developed from 11 genomes shows the lines of evolutionary descent relationship among *C. speciosa* and 10 other species. The numbers at branch nodes indicate the divergence time. The pie charts inserted in each branch show the relative genome size expansion (the right-side color block) and contraction (the left-side color block). The numbers of gene family expanded (+) and contracted (−) in each plant species were placed on the right.

To understand the evolution of the *C. speciosa* genome, orthologous clustering was completed with *C. speciosa*, *Vitis vinifera*, *Carica papaya*, *Arabidopsis thaliana*, and seven representative species of Rosaceae (*Malus domestica*, *Prunus armeniaca*, *Prunus persica*, *Prunus yedoensis*, *Pyrus betulifolia*, *Pyrus communis,* and *Rosa chinensis*). According to sequence similarity, all protein-coding genes were clustered into 6968 gene families, which were commonly identified in these 11 species. Meanwhile, 5658 specific gene families were identified in the *C. speciosa* genome (Fig. 2A). Biological process analysis via GO enrichment showed that these specific genes were primarily involved in cofactor metabolic, coenzyme metabolic, and methionine metabolic processes. The molecular function analysis via GO showed that they were involved in endonuclease activity, pyruvate kinase activity, and potassium ion binding (Fig. S17, see online supplementary material). Meanwhile, KEGG enrichment analysis showed that the species-specific genes were mainly involved in metabolic processes, including carbon metabolism, pyruvate metabolism, galactose metabolism, and terpenoid backbone biosynthesis (Fig. S18, see online supplementary material). These results suggested that *C. speciosa* had evolved unique genetic and molecular mechanisms associated with the biosynthesis of primary and secondary metabolites.

The phylogenetic status of the *C. speciosa* genome was analysed by using 10 other plant genomes (Fig. 2C). This analysis obtained 140 single-copy orthogroups identified from the 11 plant genomes. The genomes of these 11 plant species and *A. thaliana* as the outgroup species were used to construct an intergenomic phylogenetic tree and estimate the divergence time. The resulting tree showed that *C. speciosa* and *M. domestica* grouped together clustered with *P. betulifolia* and *P. communis*. In taxonomy, these four species belong to the Maleae tribe of the Amygdaloideae, indicating that their genomes have likely evolved from the same ancient ancestor. *P. armeniaca*, *P. persica*, and *P. yedoensis*, which belong to the Amygdaleae tribe of the Amygdaloideae, were clustered together. *R. chinensis*, a member of the Roseae tribe of the Rosoideae, was separated from those species in the Amygdaloideae. These results showed that the topological positions of these plants in the phylogenetic tree were consistent with their botanic phylogeny. The divergent time between *C. speciosa* and *M. domestica* occurred ~10.0 million years ago (MYA). The Maleae tribe arose from the ancestor of the Amygdaleae tribe ~10.92 MYA. The Rosaceae species diverged ~98.81 MYA.

### Expansion and contraction of gene families

To understand lineage-specific dynamic changes in the genome, annotations were mined to identify gene families that were significantly expanded or contracted (Fig. 2C). The results identified 158 contracted gene families and 320 expanded gene families in the *C. speciosa* genome. These data showed that the numbers of expanded gene families in *C. speciosa* and *M. domestica* genomes were similar. In addition, compared with other species in the Rosaceae, the number of expanded families was higher (Figs S19 andS20, see online supplementary material). By contrast, the number of contracted gene families in *C. speciosa* was ~50% of those in *M. domestica*. GO enrichment analysis showed that the contracted gene families were involved in ‘lipid transport’ and ‘signal transduction’ of the biological process category and ‘ADP binding’ and ‘squalene monooxygenase activity’ included in the molecular function category (Fig. S21, see online supplementary material). KEGG analysis revealed that the contracted gene families were associated with cyanoamino acid metabolism, steroid biosynthesis, phenylpropanoid biosynthesis, sesquiterpenoid and triterpenoid biosynthesis, starch and sucrose metabolism, and biosynthesis of tropane, piperidine, and pyridine alkaloid (Fig. S22, see online supplementary material). Meanwhile, KEGG enrichment analysis revealed that the expanded gene families were associated with the biosynthesis of monoterpenoids, stilbenoids, diarylheptanoids, gingerol, linoleic acid, flavonoids, sesquiterpenoids, and triterpenoids (Fig. 2B).

### Amplification of transposable elements

**Figure 3:**
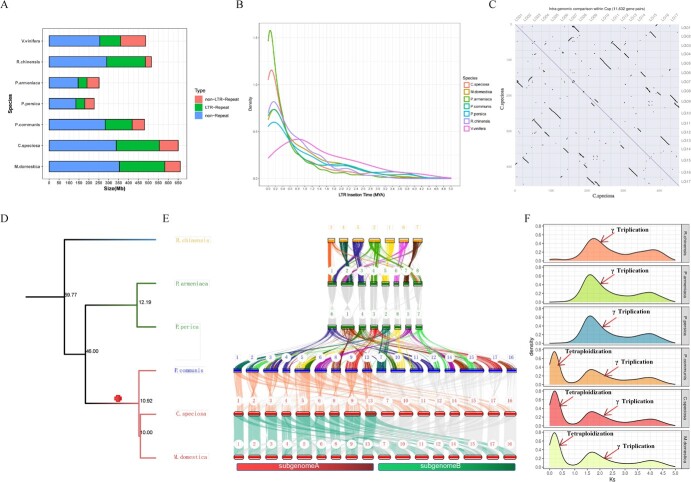
Features of whole genome duplication (WGD), synteny, and chromosome evolution of *Chaenomeles speciosa*. (**A**) The contents of repetitive sequences were compared in the genomes of *Vitis vinifera*, *Rosa chinensis*, *Prunus armeniaca*, *Prunus persica*, *Pyrus communis*, *C. speciosa*, and *Malus domestica.* (**B**) The LTR insertion times were compared in *V. vinifera*, *R. chinensis*, *P. armeniaca*, *P. persica*, *P. communis*, *C. speciosa*, and *M. domestica*. (**C**) A plot shows features resulted from self-collinearity analysis of *C. speciosa*. (**D**) A phylogenetic tree was developed with six species in Rosaceae. (**E**) A diagram was created from the intergenomic co-linearity analysis of *R. chinensis*, *P. armeniaca*, *P. persica*, *P. communis*, *C. speciosa*, *M. domestica*. (**F**) Plots show the synonymous substitutions per synonymous site (*Ks*) distributions of *R. chinensis*, *P. armeniaca*, *P. persica*, *P. communis*, *C. speciosa*, and *M. domestica*.

Sequence mining disclosed that TEs were one main event in the expansion of the genome size of *C. speciosa*. To understand the roles of TEs in this expansion, the TE content of *V. vinifera*, *R. chinensis*, *P. armeniaca*, *P. persica*, *P. communis*, *C. speciosa*, and *M. domestica* were determined for comparison (Fig. 3A). Based on the types of TE, the genomes were classified into non-repeat, LTR-repeat, and non-LTR-repeat types. The results showed that the genomes of apple and *C. speciosa* had a similar LTR-repeat and non-LTR-repeat content that expanded during their evolution. In contrast, the LTR-repeat and non-LTR-repeat contents were contracted in the *P. armeniaca* and *P. persica* genomes. Further analysis of the insertion times of LTRs indicated that the proliferation of LTRs was ~0.05 and 0.08 MYA in *P. armeniaca* and *C. speciosa*, respectively and ~ 0.15 MYA in *R. chinensis*, pear, apple, and peach (Fig. 3B). This analysis also disclosed that the insertion time of LTRs was much earlier in grape than in the other six species.

**Figure 4:**
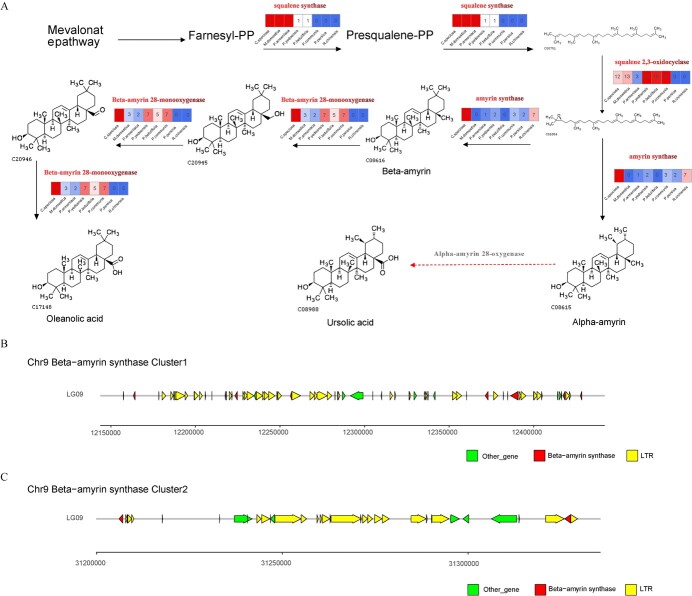
Proposed biosynthetic pathways of oleanolic acid and ursolic acid and beta-amyrin synthase gene clusters in *Chaenomeles speciosa.* (**A**) Numbers of genes encoding enzymes of the biosynthetic pathway of oleanolic acid and ursolic acid in eight plants. Those Arabic numerals included in each box above each arrow are member numbers in each gene family encoding the enzyme in *Malus domestica*, *Prunus armeniaca*, *Prunus persica*, *Prunus yedoensis*, *Pyrus betulifolia*, *Pyrus communis, Rosa chinensis*, and *C. speciosa.* (**B**) and (**C**) Distribution of two beta-amyrin synthase gene clusters on *C. speciosa* chromosome 9.

### WGD and synteny analysis

WGD was another main event in the expansion of the genome size of *C. speciosa*. Self-collinearity analysis was performed to characterize synteny and WGD in the *C. speciosa* genome (Fig. 3C). The resulting data revealed syntenic chromosome pairs, such as LG01 and LG07, LG02 and LG15, LG03 and LG11, LG04 and LG12, LG05 and LG10, LG06 and LG14, LG08 and LG15, and LG09 and LG17. These results indicated that WGD occurred in the *C. speciosa* genome. To understand WGD, six genomes from six Rosaceae species were used for chromosome evolution analysis (Fig. 3D and E). The results from the intergenomic collinearity analysis revealed a potential karyotype rearrangement between *R. chinensis* and *P. armeniaca*. The arrangements were characterized by a syntenic relationship between chromosomes 1 and 8 of *P. armeniaca* and chromosome 3 of *R. chinensis*, between chromosomes 1 and 3 of *P. armeniaca* and chromosome 5 of *R. chinensis*, between chromosomes 2 and 6 of *P. armeniaca* and chromosome 6 of *R. chinensis,* and between chromosomes 2 and 7 of *P. armeniaca* and chromosome 7 of *R. chinensis* (Fig. 3E). It was interesting that the clades resulted from the synteny analysis indicated a 1:1 syntenic relationship between *P. armeniaca* and *P. perica* (Fig. 3D and E). Meanwhile, the resulting syntenic relation cluster showed that *P. communis, M. domestica,* and *C. speciosa* were located in the clade and diverged ~46 MYA. This datum suggested that these three species underwent tetraploidization leading to doubling of their chromosomes to 16. To further characterize those orthologous chromosome and gene pairs, the synonymous substitutions per synonymous site (*Ks*) were calculated for the six species. The resulting data showed a common peak that occurred at 4.2–4.3 in the *Ks* distributions plot of these plants and indicated that four species likely underwent a eudicot-specific WGD event (Fig. 3F). Moreover, a prominent peak of the *Ks* distribution was observed at 0.2–0.3 in the genomes of *C. speciosa*, *M. domestica*, and *P. communis* (Fig. 3F). This result suggested that this event was associated with a recent tetraploidization event in *P. communis*, *C. speciosa*, and *M. domestica*.

### Construction of the metabolic pathway of oleanolic acid and ursolic acid

Oleanolic acid and ursolic acid are two pentacyclic triterpenoids, which have been specified by the Chinese Pharmacopoeia to be characteristic chemical markers of *C. speciosa* associated with medicinal applications. Gene annotation and sequences were mined to obtain candidate genes involved in the biosynthesis of the two compounds in *C. speciosa* (Fig. 4A). The candidate genes allowed the construction of the biosynthetic steps from the mevalonate pathway to the two compounds (Fig. 4A). To characterize genes specifically associated with steps starting with farnesyl diphosphate, the size of four gene families (squalene synthase, squalene 2,3-oxidocyclase, amyrin synthase, and beta-amyrin 28-monoxygenase) was compared in *C. speciosa* and seven other species (Fig. 4A). The resulting data determined 11 beta-amyrin synthase genes found in the genome of *C. speciosa*, 10 of which were distributed in two gene clusters on chromosome 9. Gene cluster 1, ranging from 12 157 281 bp to 12 428 418 bp, contained eight beta-amyrin synthase genes, nine other genes, and 64 LTR retrotransposons (Fig. 4B). Gene cluster 2, ranging from 31 206 028 bp to 31 327 645 bp, included two beta-amyrin synthase genes, five other genes, and 29 LTR retrotransposons (Fig. 4C). It was interesting that the gene family encoding alpha amyrin 28-oxygenases was not found in any of the genomes examined. This warrants further investigation. The characterization of gene families further revealed two squalene synthase members, 12 squalene 2,3-oxidocyclase members, 11 beta-amyrin synthase members, and nine beta-amyrin 28-monooxygenase members in the *C. speciosa* genome. Compared with the other Rosaceae species examined, the squalene synthase, beta-amyrin synthase, and beta-amyrin 28-monooxygenase gene families were obviously expanded in the *C. speciosa* genome, associated with the high oleanolic acid and ursolic acid content in this species.

## Discussion

Our genome sequencing and assembly provide valuable information for promoting genetic breeding efforts and cultivation of *C. speciosa* for improved use. This plant is not only an ornamental crop, but also a medicinal and edible plant with significant economic value that is cultivated all over the world. However, its genetic breeding has been relatively slow, mainly because our understanding of its genetics is limited. In addition, none of the five species of *Chaenomeles* have been characterized in terms of their genomes, molecular biology, and so on. The long-read sequencing technologies, assembly algorithms, and T2T have been reported to be excellent approaches for assembling high-quality genomes for crop plants, such as rice [15] and watermelon [16]. In this report, we used these technologies to develop a nearly gap-free T2T genome for *C. speciosa*, which revealed it to have a relatively high heterozygosity. The *k*-mer analysis showed that the heterozygosity rate of the *C. speciosa* genome was 2.1%, which was significantly higher than that in the genomes of pear (0.89%) [20], apple (0.85–1.28%), peach (0.31%) [21], loquat (0.31%) [22], *Prunus mume* (0.75%) [23], hawthorn (1.77%) [19], and plum (0.92%). In addition, the chromosome-level genome of *C. speciosa* assembled with its 632.3 Mb sequence and a contig N50 of 32.3 Mb is valuable for planning of future breeding efforts, enabling a comparison of its complexity with other important crops in the Rosaceae, which have been sequenced for genetic breeding. Our assembly showed that the genome size of *C. speciosa* was comparable to that of apple (652–668 Mb) [18, 24], smaller than that of hawthorn (779.24 Mb) [19], and loquat (733.32 Mb), and larger than that of pear (496.9–541.34 Mb) [ 20, 25, 26]. It was interesting that WGD events had occurred in the genome of *C. speciosa*, but did not occur in these species. The contracted content of LTR was lower in *C. speciosa* than in apple. More importantly, the resulting T2T genome included 11 chromosomes with telomere repeats at both ends and four chromosomes with telomere repeats at a single end. Thus, our T2T genome provided an important source to comparatively understand genome features in the Rosaceae, given that the assembly of T2T genomes can help understand whether the telomere repeats prevail in other plant species. On the one hand, in an apple T2T genome assembled recently, telomeres were assembled at two ends of seven chromosomes and at a single end of eight chromosomes [24]. A T2T genome assembled from a wild-type pear showed that telomere repeats were also detected at both ends of five chromosomes and at a single end of eight chromosomes [25]. On the other hand, no telomere repeats were reported in the genomes assembled for hawthorn [19], loquat [22], and cultivated pear [24, 27], indicating the need for continuous genome assembly to understand plant genome evolution in the Rosaceae.

Compared with other plants, the T2T genome assembly for *C. speciosa* revealed 12 pseudo-chromosomes represented by a single contig, four pseudo-chromosomes characterized by two contigs, and one pseudo-chromosome characterized by four contigs. These profiles suggested that seven gaps existed in the assembly of the *C. speciosa* genome. Preliminary analysis indicated that those gaps could not be assembled continuously because of the existence of large fragments of dispersed repeats and inverted repeats in the gap-flaking region. Moreover, our T2T genome was characterized by high values of contig N50, BUSCO, LAI, and short-read mapping ratios. These results indicated the high contiguity, completeness, and accuracy of the *C. speciosa* genome assembled in this study. Based on the application of genome sequencing in the breeding of apple and other crops for improved economic values, it can be anticipated that the genome assembled in this study will be useful for breeding efforts to develop new value-added *C. speciosa* cultivars in the future.

Orthologous clustering analysis enhances the identification of species-specific gene families associated with different biological or metabolic processes in plants in the Rosaceae. Enrichment analysis revealed the species-specific gene families of loquat, which were characterized to associate with the evolution of unique genetic and molecular mechanisms for genome recombination and repair [22]. Another enrichment analysis disclosed that species-specific gene families of hawthorn were involved in biosynthetic and metabolic processes, such as starch and sucrose metabolism and fatty acid degradation [19]. The lineage-specific gene families of pear were reported to associate with DNA metabolic processes, DNA integration, DNA recombination, and cellulose microfibril organization [25]. Our enrichment analysis revealed that multiple specific gene families of *C. speciosa* were mainly involved in metabolic processes, such as galactose metabolism and terpenoid backbone biosynthesis. In addition, conserved domain enrichment analysis of the specific genes of Hap1 and Hap2 showed that they were enriched to some conserved domains related to reverse transcriptase, suggesting the origin of these specific genes from the transposition of retrotransposons. Our assembly estimated gene families that were expanded or contracted in the *C. speciosa* genome. Such expansion and contraction have important roles in the functional diversification of genes in plants of the Rosaceae. It was reported that the expanded gene families in the hawthorn genome were involved in biosynthetic pathways of plant natural products [19]. In the loquat genome, expanded gene families were found to involve monoterpenoid biosynthesis and starch and sucrose metabolism [22]. Our functional annotation of genes found that the number of the expanded gene families was nearly twofold more than that of contracted ones in the *C. speciosa* genome. These expanded gene families were involved in monoterpenoid biosynthesis, flavonoid biosynthesis, sesquiterpenoid and triterpenoid biosynthesis, and others. Beta-amyrin synthase was more redundant in *C. speciosa* than in other Rosaceae species sequenced, suggesting that LTR mediates the proximal duplication of such enzymes on the genome and the formation of two beta-amyrin synthase gene clusters on chromosome 9. By analysing other genomes, we found that *R. chinensis* had one beta-amyrin synthase gene cluster containing five beta-amyrin synthase on chromosome 2. It is interesting that all other Rosaceae plants studied to date did not have beta-amyrin synthase gene clusters, although they had between two and four members of this gene family.

Based on these results, we further examined gene families involved in the biosynthesis of health-promoting triterpenoids in *C. speciosa* fruits [1, 7, 28]. The main pathway gene families were identified to associate with the biosynthesis of oleanolic and ursolic acids in *C. speciosa* (Fig. 4A). Although the functions of these gene families in *C. speciosa* require further studies, the annotation of them, such as of alpha-amyrin synthase and beta-amyrin synthase enzymes in apple, supported our functional annotations [29]. Furthermore, the expansion of two gene families was higher in the *C. speciosa* genome than in other plants in the Rosaceae. These data supported the high production of oleanolic and ursolic acids in this species and, thus, are valuable for further elucidation of plant secondary metabolism in *C. speciosa*.

## Conclusions

A T2T and chromosome-level genome was assembled for *C. speciosa*. This is the first plant genome assembled from the *Chaenomeles* genus in the Rosaceae. This high-quality genome featured high heterozygosity and was 650.4 Mb in size. The genome was anchored to 17 pseudo-chromosomes with a contig N50 of 35.5 Mb and a scaffold N50 of 35.8 Mb. Twelve pseudo-chromosomes were represented by single contigs, and seven gaps existed in the assembly. Eleven pseudo-chromosomes had telomere repeats at both ends, and four had telomere repeats at single ends. Additionally, predictions were made for the centromere regions on all pseudo-chromosomes in the *C. speciosa* genome. In total, 45 515 protein-coding genes were annotated. Species-specific gene families that were expanded or contracted were also identified. Functional annotation indicated that mainly expanded gene families were associated with plant secondary metabolism, which enabled the construction of the biosynthetic pathway of medicinal oleanolic acid and ursolic acid. LTR mediated multiple proximal replications of beta-amyrin synthase genes in the genome of *C. speciosa*, which had more members compared with other Rosaceae plants sequenced thus far. The high-quality genome assembled formed a valuable platform for investigating the biosynthesis and metabolic engineering of medicinal compounds in *C. speciosa*.

## Materials and methods

### Plant materials

*C. speciosa* is a diploid shrub, numerous plants of which have been cultivated for research in the research station on our campus in Wuhan, Hubei Province, China. Its healthy leaves, stems, roots, flowers, and fruits were collected in liquid nitrogen and stored at −80°C in a freezer until use.

### High-fidelity sequencing and genome assembly

Genomic DNA was extracted from leaves using a Plant Genomic DNA kit (Tiangen, Beijing, China) for HiFi sequencing, which is a third-generation sequencing technology. A SMRTbell Express Template Prep Kit 2.0 (Pacific Biosciences of California, Menlo Park, CA, USA) was used to construct a Single Molecule Real-Time (SMRT) sequencing library, and the library was sequenced on a PacBio Sequel II System with one 8 M SMRT cell. To obtain the HiFi reads, the subreads were processed with ccs software (https://github.com/PacificBiosciences/ccs). The genome size, heterozygosity, and proportion of repetitive sequences were estimated with K-mer analysis (*k* = 21) by jellyfish (https://github.com/gmarcais/Jellyfish) and GCE (https://github.com/fanagislab/GCE). High-quality HiFi reads were then assembled into contigs using Hifiasm with default haplotig purging parameters and ‘–primary’ [30]. Additionally, haploid assembly was performed using the parameter ‘Hi-C-partition: -hl/2’, and Hic sequencing data were added for haploid assembly and typing.

### Detection of haploid variation

For haploid SNP-InDel detection, we performed collinearity alignment using nucmer (—mum, —maxgap = 500, —mincluster = 100) and filtered the results using the delta-filter (−1, −q, −r) program. We then used show-snps to input the SNP and InDel sites, with these programs being subroutines of MUMmer (v4.0). To convert the SNP and InDel site files into vcf format files, we used the MUMmerSNPs2VCF.py script. SV between two haploids was detected using MummandCo (V3.0) with default parameters, and the collinear graph of the two haploids was drawn using GenomeSyn-1.2.7.

### Chromosome conformation capture sequencing and scaffolding

The chromosome conformation capture (Hi-C) sequencing method was utilized to generate paired-end reads of 150 bp after chromatin digestion using MboI. FastQC (www.bioinformatics.babraham.ac.uk/projects/fastqc/) was used to assess the quality of reads, and fastp [31] was used to filter them. Valid read pairs were identified using HiCUP (www.bioinformatics.babraham.ac.uk/projects/hicup/), and BWA was used to align them to contigs. Juicer 1.6 was utilized to generate chromosomal contact frequency maps, which were then used for scaffolding using the 3D-DNA pipeline (https://github.com/aidenlab/3d-dna). The scaffolds were manually corrected using Juicebox Assembly Tools (https://github.com/aiden lab/Juicebox). The genome assembly completeness was evaluated using BUSCO analysis. D-GENIES was used to align scaffolds to the apple reference genome to determine their identities.

### Iso-seq sequencing

RNA samples were extracted from different plant tissues using TRIzol reagent (Invitrogen, Carlsbad, CA, USA). The cDNA synthesis was synthesized using a SMARTer™ PCR cDNA Synthesis Kit (Takara Biotechnology, Dalian, Liaoning, China). SMRTbell libraries were constructed using a Pacific Biosciences DNA Template Prep Kit 2.0 and sequenced on a PacBio Sequel II platform by Frasergen Bioinformatics Co., Ltd (Frasergen, Wuhan, Hubei, China).

### Centromere and telomere identification

Telomere sequence identification was carried out by examining the presence of repetitive sequences (TTAGG) in the telomeric region. Centromere identification was performed using the Centromics17 program, utilizing genome sequence data, raw HiFi sequencing data, and Hi-C sequencing data as inputs.

### Repetitive element identification

Tandem repeats were identified and soft-masked using the Tandem Repeats Finder with default parameters and BEDTools [32]. Repeat analysis was conducted through a homology-based approach and *ab initio* prediction. Homologous sequences were detected using RepeatMasker (www.repeatmasker.org) based on the RepBase (v.21.12) library (www.girinst.org/repbase), and LTR-RT were identified using LTR_Finder according to a protocol reported previously [33]. RepeatModeler (www.repeatmasker.org/RepeatModeler/) was used to build the *ab initio* prediction repeat library. The LTRs and *de novo* repeat library were combined to screen the genome assembly using RepeatMasker. The assembly completeness was evaluated by calculating the LTR assembly index score from the EDTA output files.

### Gene prediction and functional annotation

To predict protein-coding genes, three approaches were used on the repeat masked genome: homology search, *de novo* prediction, and Iso-seq data. The gene models were predicted using the BRAKER2 pipeline [34], and their functions were annotated based on alignments to genes in the non-redundant protein sequences (nr) of NCBI, TrEMBL, BLASTP of InterPro and Swiss-Prot protein databases, and KEGG database, with an E-value threshold of <1E-5. Protein domains were annotated using PfamScan and InterProScan, and motifs and domains within gene models were identified using the PFAM database. Gene Ontology IDs were annotated using Blast2GO.

Additionally, noncoding RNA genes were annotated using various tools. tRNAs were predicted using tRNAscan-SE (https://github.com/UCSC-LoweLab/tRNAscan-SE) with eukaryote parameters, whereas mRNA, snRNA, and snoRNA were predicted using INFERNAL (http://eddylab.org/infernal/) based on the Rfam and miRbase databases. The rRNAs and their subunits were predicted using RNAmmer (http://cbs.dtu.dk/services/RNAmmer/).

### Gene family identification and phylogenetic analysis

To construct a phylogenetic tree, the genomes of 11 species were used: *C. speciosa*, *M. domestica, P. armeniaca, P. persica*, *P. yedoensis*, *P. betulifolia*, *P. communis*, *R. chinensis*, *C. papaya, V. vinifera*, and *A. thaliana* (used as the outgroup). NCBI was the source for downloading the genome-wide protein sequences for each species. OrthoFinder [35] was used to construct gene families for the 11 species, and single-copy orthogroups were identified for establishing a phylogenetic tree. Amino acid alignments were performed for each single-copy orthogroup using MAFFT [36], whereas nucleotide alignments were created using PAL2NAL, which can be accessed at www.bork.embl.de/pal2nal/. A maximum likelihood phylogeny was then established using IQ-TREE [37] based on the concatenated alignments of all single-copy genes. MCMCTREE in PAML [38] was utilized to assess the species divergence time. Finally, gene family expansion and contraction were explored using CAFE [39].

### Whole-genome duplication analysis

To analyse the WGD event, we selected the genomes of *C. speciosa*, *M. domestica*, *P. communis*, *P. armeniaca*, *R. chinensis*, and *P. persica.* We determined the WGD events by calculating the synonymous substitutions per synonymous site [20] in the genome. BlastP with a cutoff of e-value <1e^−5^ was used to identify homologs in each genome. Protein-coding DNA alignments were performed using ParaAT for the homologs (https://ngdc.cncb.ac.cn/tools/paraat). Ks values were then calculated using KaKs_Calculator (https://ngdc.cncb.ac.cn/tools/kaks). To plot the *Ks* distributions, we used the ggplot2 package from R.

## Supplementary Material

Web_Material_uhad183

## Data Availability

The whole genome sequence data reported in this paper have been deposited in the NCBI BioProject under accession number PRJNA890604 and NCBI BioSample under accession number SAMN31283309. The genome data referred in this article: *Rosa chinensis* (NCBI: GCF_002994745.2), *Prunus armeniaca* (NCBI: GCA_020424065.1), *Prunus persica* (NCBI: GCA_000346465.2), *Pyrus communis* BartlettDHv2.0 (PyrusCommunis_BartlettDHv2.0, http://peargenome.njau.edu.cn/), *Malus domestica* (NCBI: GCA_004115385.1), *Prunus yedoensis* (NCBI: GCA_002966975.2), *Carica papaya* (NCBI:GCF_000150535.2), *Vitis vinifera* (NCBI: GCA_000003745.2), *Pyrus betulifolia* (NGDC:GWHAAYT00000000), *Arabidopsis thaliana* (NCBI: GCF_000001735.4).

## References

[1] Tao W , Zhao C, Lin G et al. UPLC-ESI-QTOF-MS/MS analysis of the phytochemical compositions from *Chaenomeles speciosa* (sweet) Nakai fruits. J Chromatogr Sci. 2022;61:15–31 35134870 10.1093/chromsci/bmac002

[2] Zhang S-Y , Han L-Y, Zhang H et al. Chaenomeles speciosa: a review of chemistry and pharmacology. Biomed Rep. 2014;2:12–8 24649061 10.3892/br.2013.193 PMC3917013

[3] Xianfei X , Xiaoqiang C, Shunying Z et al. Chemical composition and antimicrobial activity of essential oils of *Chaenomeles speciosa* from China. Food Chem. 2007;100:1312–5

[4] Huang W , He J, Nisar MF et al. Phytochemical and pharmacological properties of *Chaenomeles speciosa*: an edible medicinal Chinese mugua. Evid Based Complement Alternat Med. 2018;2018:9591845 30622618 10.1155/2018/9591845 PMC6304597

[5] Miao J , Zhao C, Li X et al. Chemical composition and bioactivities of two common Chaenomeles fruits in China: *Chaenomeles speciosa* and *Chaenomeles sinensis*. J Food Sci. 2016;81:H2049–58 27384225 10.1111/1750-3841.13377

[6] Xie X , Zou G, Li C. Purification, characterization and in vitro antioxidant activities of polysaccharide from Chaenomeles speciosa. Int J Biol Macromol. 2016;92:702–7 27471089 10.1016/j.ijbiomac.2016.07.086

[7] Ma Y , Li J, Li J et al. Comparative metabolomics study of *Chaenomeles speciosa* (sweet) Nakai from different geographical regions. Foods. 2022;11:1019 35407106 10.3390/foods11071019 PMC8997580

[8] Zhang L , Cheng YX, Liu AL et al. Antioxidant, anti-inflammatory and anti-influenza properties of components from *Chaenomeles speciosa*. Molecules. 2010;15:8507–17 21102377 10.3390/molecules15118507 PMC6259204

[9] Chen K , You J, Tang Y et al. Supplementation of superfine powder prepared from *Chaenomeles speciosa* fruit increases endurance capacity in rats via antioxidant and Nrf2/ARE signaling pathway. Evid Based Complement Alternat Med. 2014;2014:1–7 10.1155/2014/976438 PMC4290570 25610489

[10] Li M , Xiao Y, Mount S et al. An atlas of genomic resources for studying Rosaceae fruits and ornamentals. Front Plant Sci. 2021;12:397 10.3389/fpls.2021.644881 PMC8047320 33868343

[11] Lawniczak MKN , Durbin R, Flicek P et al. Standards recommendations for the earth BioGenome project. Proc Natl Acad Sci U S A. 2022;119:e2115639118 35042802 10.1073/pnas.2115639118 PMC8795494

[12] Sun Y , Shang L, Zhu QH et al. Twenty years of plant genome sequencing: achievements and challenges. Trends Plant Sci. 2022;27:391–401 34782248 10.1016/j.tplants.2021.10.006

[13] Shang X , Yi X, Xiao L et al. Chromosomal-level genome and multi-omics dataset of *Pueraria lobata* var. thomsonii provide new insights into legume family and the isoflavone and puerarin biosynthesis pathways. Hortic Res. 2022;9:uhab035 35043180 10.1093/hr/uhab035 PMC8881381

[14] Mo C , Wu Z, Shang X et al. Chromosome-level and graphic genomes provide insights into metabolism of bioactive metabolites and cold-adaption of *Pueraria lobata* var. montana. DNA Res. 2022;29:1–11 10.1093/dnares/dsac030 PMC9397507 35961033

[15] Zhang Y , Fu J, Wang K et al. The telomere-to-telomere gap-free genome of four rice parents reveals SV and PAV patterns in hybrid rice breeding. Plant Biotechnol J. 2022;20:1642–4 35748695 10.1111/pbi.13880 PMC9398309

[16] Deng Y , Liu S, Zhang Y et al. A telomere-to-telomere gap-free reference genome of watermelon and its mutation library provide important resources for gene discovery and breeding. Mol Plant. 2022;15:1268–84 35746868 10.1016/j.molp.2022.06.010

[17] Nie S , Zhao S-W, Shi T-L et al. Gapless genome assembly of azalea and multi-omics investigation into divergence between two species with distinct flower color. Hortic Res. 2023;10:10 10.1093/hr/uhac241 PMC9832866 36643737

[18] Sun X , Jiao C, Schwaninger H et al. Phased diploid genome assemblies and pan-genomes provide insights into the genetic history of apple domestication. Nat Genet. 2020;52:1423–32 33139952 10.1038/s41588-020-00723-9 PMC7728601

[19] Zhang T , Qiao Q, Du X et al. Cultivated hawthorn (*Crataegus pinnatifida* var. major) genome sheds light on the evolution of Maleae (apple tribe). J Integr Plant Biol. 2022;64:1487–501 35748532 10.1111/jipb.13318

[20] Gao Y , Yang Q, Yan X et al. High-quality genome assembly of ‘Cuiguan’ pear (*Pyrus pyrifolia*) as a reference genome for identifying regulatory genes and epigenetic modifications responsible for bud dormancy. Hortic Res. 2021;8:197 34465760 10.1038/s41438-021-00632-w PMC8408243

[21] Lian X , Zhang H, Jiang C et al. De novo chromosome-level genome of a semi-dwarf cultivar of *Prunus persica* identifies the aquaporin PpTIP2 as responsible for temperature-sensitive semi-dwarf trait and PpB3-1 for flower type and size. Plant Biotechnol J. 2022;20:886–902 34919780 10.1111/pbi.13767 PMC9055816

[22] Wang Y . A draft genome, resequencing, and metabolomes reveal the genetic background and molecular basis of the nutritional and medicinal properties of loquat (*Eriobotrya japonica* (Thunb.) Lindl). Hortic Res. 2021;8:231 34719689 10.1038/s41438-021-00657-1 PMC8558328

[23] Zheng T , Li P, Zhuo X et al. The chromosome-level genome provides insight into the molecular mechanism underlying the tortuous-branch phenotype of *Prunus mume*. New Phytol. 2022;235:141–56 34861048 10.1111/nph.17894 PMC9299681

[24] Zhang L , Hu J, Han X et al. A high-quality apple genome assembly reveals the association of a retrotransposon and red fruit colour. Nat Commun. 2019;10:1–13 30940818 10.1038/s41467-019-09518-x PMC6445120

[25] Dong X , Wang Z, Tian L et al. De novo assembly of a wild pear (*Pyrus betuleafolia) genome*. Plant Biotechnol J. 2020;18:581–95 31368610 10.1111/pbi.13226 PMC6953202

[26] Linsmith G , Rombauts S, Montanari S et al. Pseudo-chromosome–length genome assembly of a double haploid ‘Bartlett’ pear (*Pyrus communis* L.). Gigascience. 2019;8:8 10.1093/gigascience/giz138 PMC6901071 31816089

[27] Shirasawa K , Itai A, Isobe S. Chromosome-scale genome assembly of Japanese pear (*Pyrus pyrifolia*) variety ‘Nijisseiki’. DNA Res. 2021;28:dsab001 33638981 10.1093/dnares/dsab001 PMC8092371

[28] Lu C , Zhang C, Zhao F et al. Biosynthesis of ursolic acid and oleanolic acid in Saccharomyces cerevisiae. AICHE J. 2018;64:3794–802

[29] Brendolise C , Yauk YK, Eberhard ED et al. An unusual plant triterpene synthase with predominant α-amyrin-producing activity identified by characterizing oxidosqualene cyclases from malus×domestica. FEBS J. 2011;278:2485–99 21575133 10.1111/j.1742-4658.2011.08175.x

[30] Cheng H , Concepcion GT, Feng X et al. Haplotype-resolved de novo assembly using phased assembly graphs with hifiasm. Nat Methods. 2021;18:170–5 33526886 10.1038/s41592-020-01056-5 PMC7961889

[31] Chen S , Zhou Y, Chen Y et al. Fastp: an ultra-fast all-in-one FASTQ preprocessor. Bioinformatics. 2018;34:i884–90 30423086 10.1093/bioinformatics/bty560 PMC6129281

[32] Benson G . Tandem repeats finder: a program to analyze DNA sequences. Nucleic Acids Res. 1999;27:573–80 9862982 10.1093/nar/27.2.573 PMC148217

[33] Xu Z , Wang H. LTR_FINDER: an efficient tool for the prediction of full-length LTR retrotransposons. Nucleic Acids Res. 2007;35:W265–8 17485477 10.1093/nar/gkm286 PMC1933203

[34] Brůna T , Hoff KJ, Lomsadze A et al. BRAKER2: automatic eukaryotic genome annotation with GeneMark-EP+ and AUGUSTUS supported by a protein database. NAR Genom Bioinform. 2021;3:1–11 10.1093/nargab/lqaa108 PMC7787252 33575650

[35] Emms DM , Kelly S. OrthoFinder: phylogenetic orthology inference for comparative genomics. Genome Biol. 2019;20:1–14 31727128 10.1186/s13059-019-1832-y PMC6857279

[36] Rozewicki J , Li S, Amada KM et al. MAFFT-DASH: integrated protein sequence and structural alignment. Nucleic Acids Res. 2019;47:W5–10 31062021 10.1093/nar/gkz342 PMC6602451

[37] Minh BQ , Schmidt HA, Chernomor O et al. IQ-TREE 2: new models and efficient methods for phylogenetic inference in the genomic era. Mol Biol Evol. 2020;37:1530–4 32011700 10.1093/molbev/msaa015 PMC7182206

[38] Yang Z . PAML 4: phylogenetic analysis by maximum likelihood. Mol Biol Evol. 2007;24:1586–91 17483113 10.1093/molbev/msm088

[39] De Bie T , Cristianini N, Demuth JP et al. CAFE: a computational tool for the study of gene family evolution. Bioinformatics. 2006;22:1269–71 16543274 10.1093/bioinformatics/btl097

