# Supplementary material for: A telomere-to-telomere reference genome provides genetic insight into the pentacyclic triterpenoid biosynthesis in Chaenomeles speciosa
Source: Hortic Res. 2023 Sep 14;10(10):uhad183. doi: 10.1093/hr/uhad183 (PMC10623406; doi:10.1093/hr/uhad183)
Supplement: Web_Material_uhad183 [file web_material_uhad183.zip › Table S11 Summary on the Hi-C scaffolding.docx]

| Pseudo-chromosome | Chromosome size | Number of contigs | Contig size | GC (%) | Left terminal | Right terminal |
| --- | --- | --- | --- | --- | --- | --- |
| LG01 | 32856102 | 1 | 32856102 | 37.94 | - | - |
| LG02 | 35454787 | 1 | 35454787 | 37.48 | Telomere | Telomere |
| LG03 | 41192068 | 1 | 41192068 | 36.29 | - | Telomere |
| LG04 | 24494482 | 1 | 24494482 | 37.62 | Telomere | Telomere |
| LG05 | 41351270 | 1 | 41351270 | 37.28 | - | Telomere |
| LG06 | 32355895 | 1 | 32355895 | 37.54 | - | - |
| LG07 | 41742070 | 2 | 41742066 | 37.31 | Telomere | Telomere |
| LG08 | 32678731 | 2 | 32678727 | 37.45 | Telomere | Telomere |
| LG09 | 50582745 | 4 | 50582733 | 37.07 | Telomere | Telomere |
| LG10 | 40833724 | 1 | 40833724 | 36.92 | Telomere | Telomere |
| LG11 | 39926357 | 1 | 39926357 | 37.4 | Telomere | Telomere |
| LG12 | 29516134 | 1 | 29516134 | 37.25 | Telomere | Telomere |
| LG13 | 35488782 | 1 | 35488782 | 37.66 | Telomere | - |
| LG14 | 32457730 | 2 | 32457726 | 36.98 | Telomere | Telomere |
| LG15 | 51822334 | 1 | 51822334 | 37.41 | Telomere | Telomere |
| LG16 | 35780769 | 1 | 35780769 | 37.75 | Telomere | Telomere |
| LG17 | 33771985 | 2 | 33771981 | 37.28 | Telomere | Telomere |
| ChrAll | 632305965 | 24 | 632305937 | 37.31 | - | - |

Table S2 Summary on the Hi-C scaffolding
