# Supplementary material for: A telomere-to-telomere reference genome provides genetic insight into the pentacyclic triterpenoid biosynthesis in Chaenomeles speciosa
Source: Hortic Res. 2023 Sep 14;10(10):uhad183. doi: 10.1093/hr/uhad183 (PMC10623406; doi:10.1093/hr/uhad183)
Supplement: Web_Material_uhad183 [file web_material_uhad183.zip › Table S22 Summary on the repetitive sequences.docx]

Table S2 Summary on the repetitive sequences

| Repeat type | Total size (bp) |
| --- | --- |
| DNA/CMC-EnSpm | 777255 |
| DNA/hAT-Ac | 506297 |
| DNA/hAT-Tag1 | 354243 |
| DNA/hAT-Tip100 | 734840 |
| DNA/MuLE-MuDR | 146861 |
| DNA/PIF-Harbinger | 3764444 |
| LINE/L1 | 675094 |
| LINE/RTE-BovB | 3618398 |
| Low_complexity | 1485587 |
| LTR/Caulimovirus | 1105279 |
| LTR/Copia | 46372495 |
| LTR/Gypsy | 143222959 |
| LTR/unknown | 38571881 |
| RC/Helitron | 477365 |
| Simple_repeat | 6587330 |
| Unknown | 87838440 |
| Total | 336238768 |
