# Supplementary figures and images for: A telomere-to-telomere reference genome provides genetic insight into the pentacyclic triterpenoid biosynthesis in Chaenomeles speciosa
Source: Hortic Res. 2023 Sep 14;10(10):uhad183. doi: 10.1093/hr/uhad183 (PMC10623406; doi:10.1093/hr/uhad183)

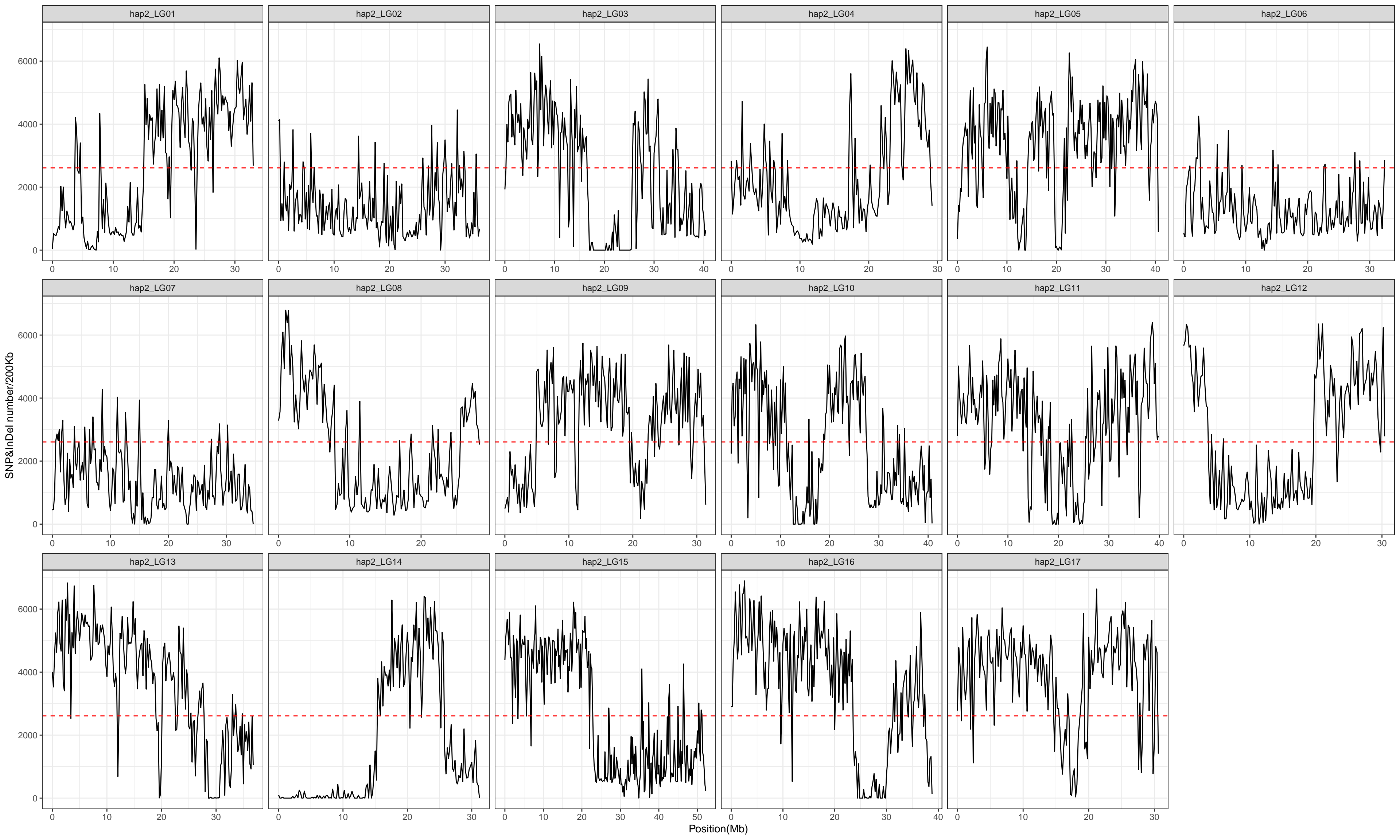

Supplement: Web_Material_uhad183 [file web_material_uhad183.zip › Fig. S1 Distribution of SNP and InDel numbers on the genome between haploids (statistics by 200 kb windows).pdf]

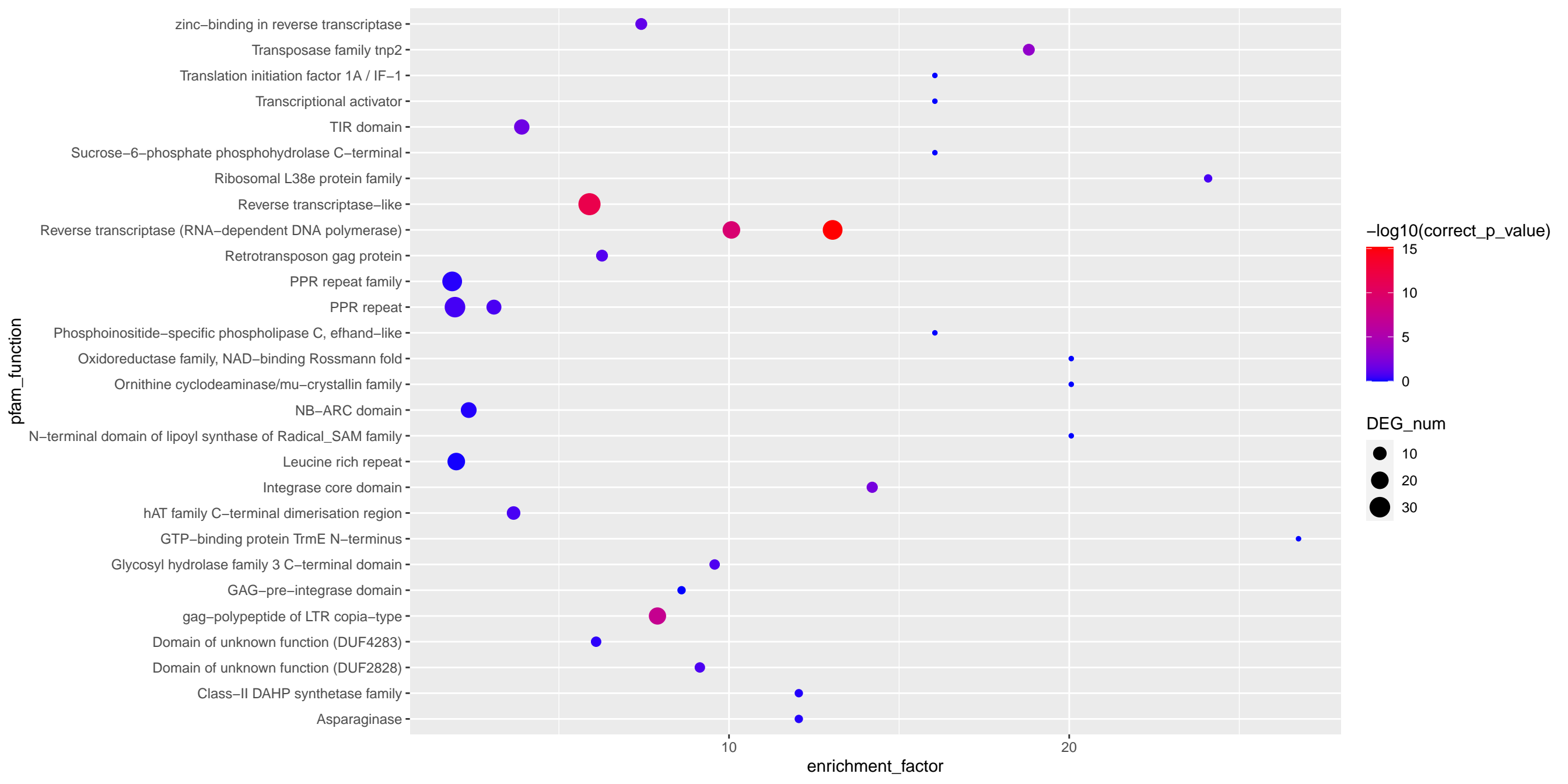

Supplement: Web_Material_uhad183 [file web_material_uhad183.zip › Fig. S10 Pfam enrichment analysis on the species-specific gene families of Hap1.pdf]

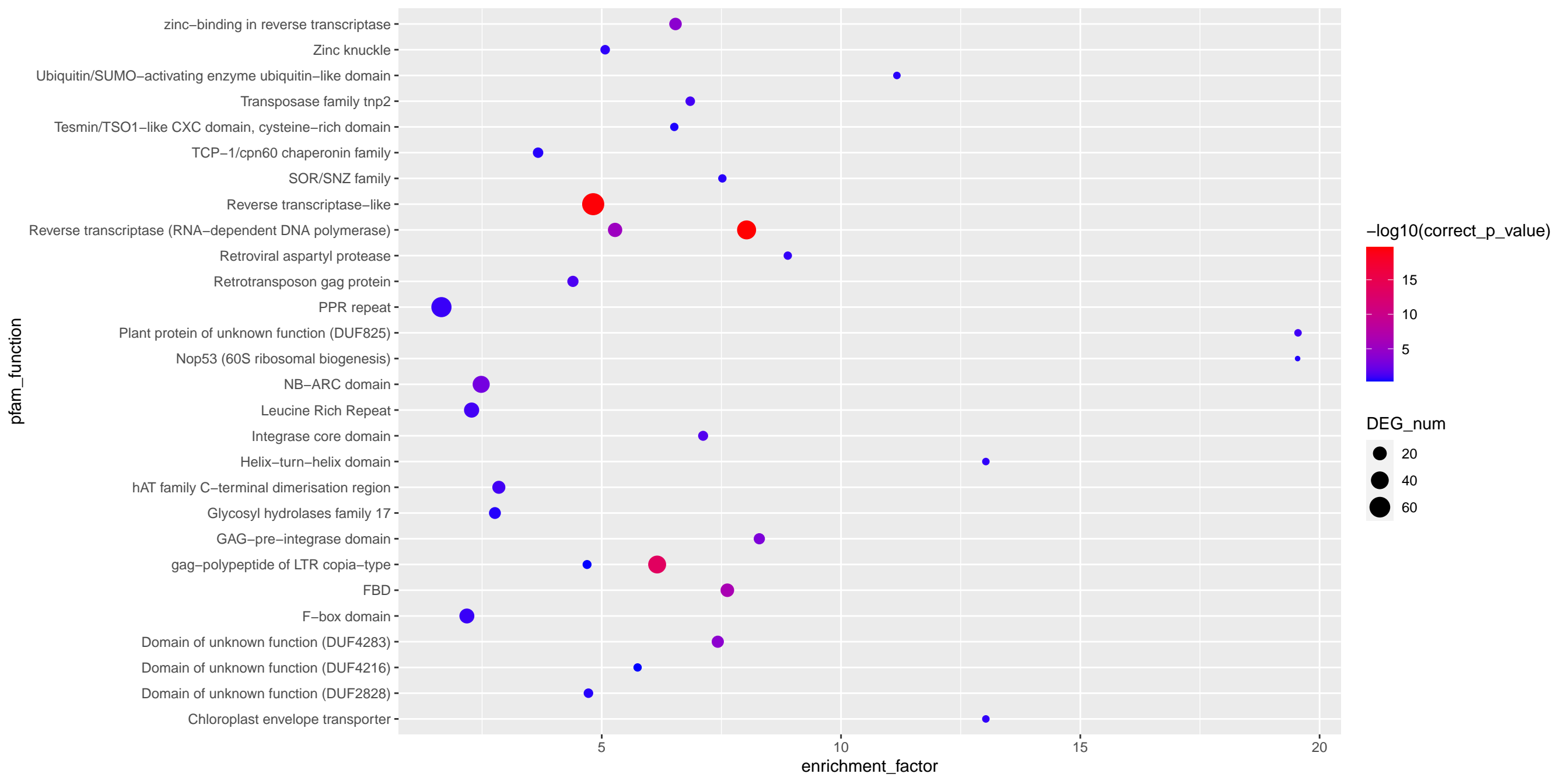

Supplement: Web_Material_uhad183 [file web_material_uhad183.zip › Fig. S11 Pfam enrichment analysis on the species-specific gene families of Hap2.pdf]

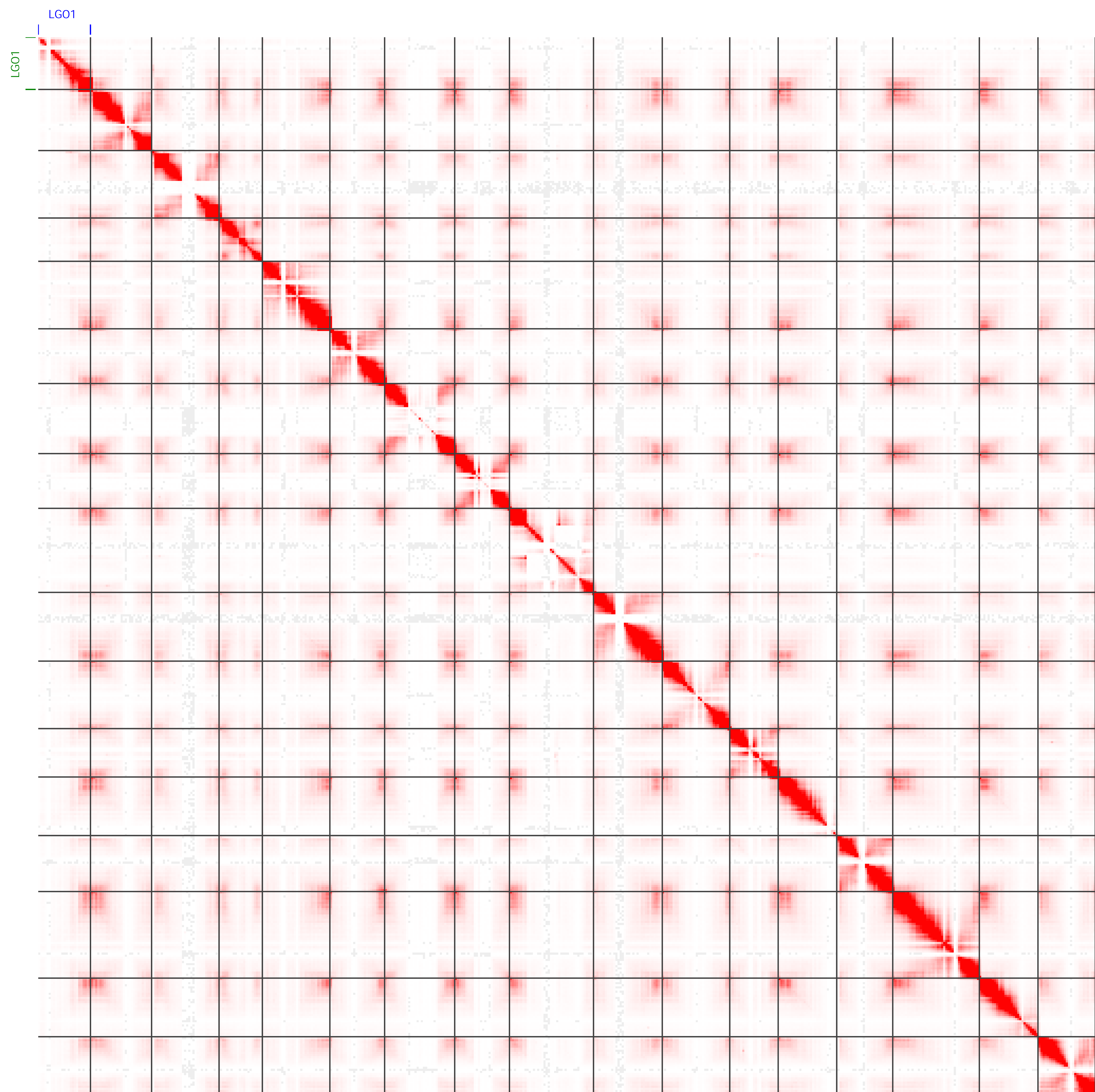

Supplement: Web_Material_uhad183 [file web_material_uhad183.zip › Fig. S12 Hi-C interaction heatmap.pdf]

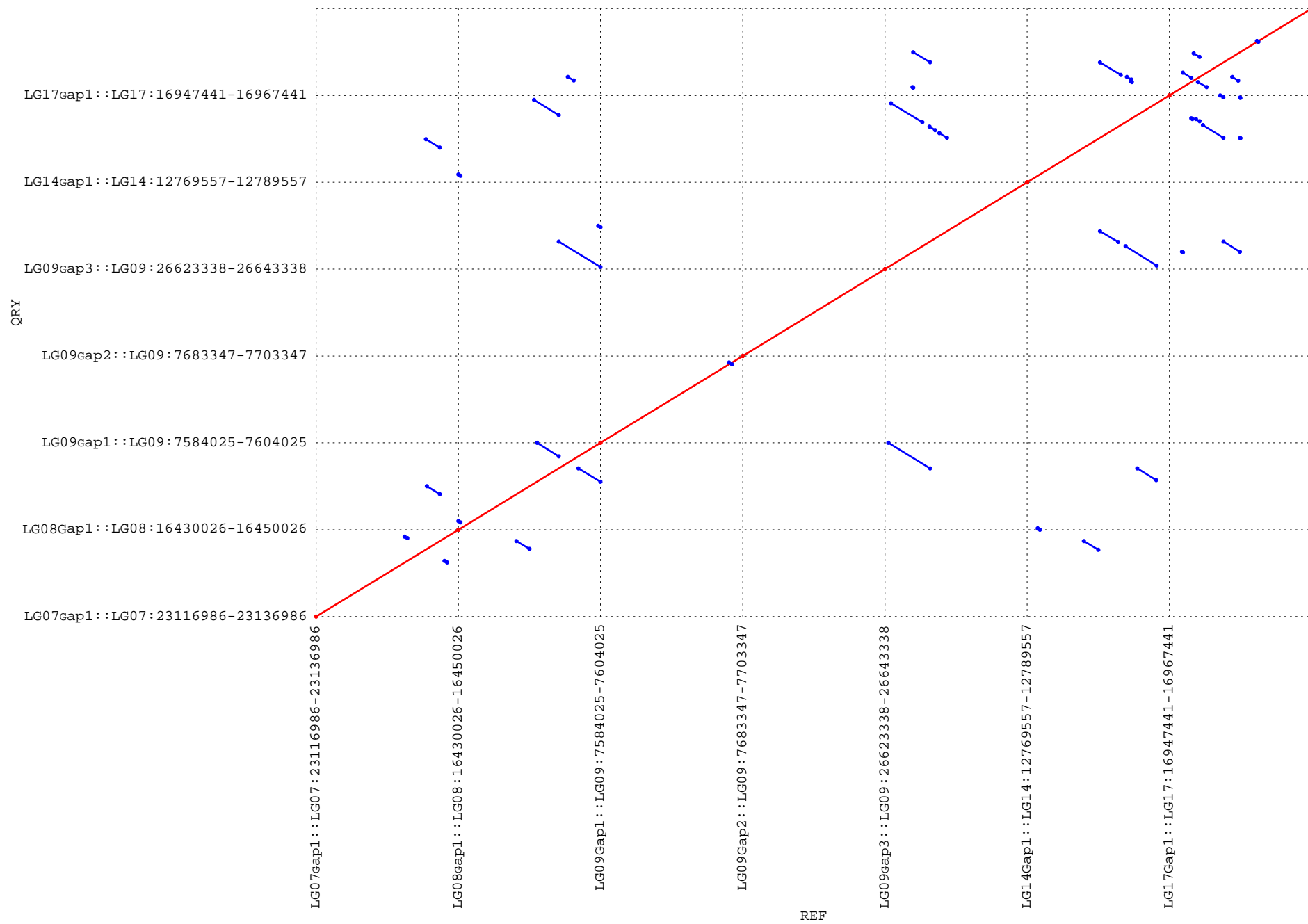

Supplement: Web_Material_uhad183 [file web_material_uhad183.zip › Fig. S14 Collinearity analysis of gap-flanking region sequence.pdf]

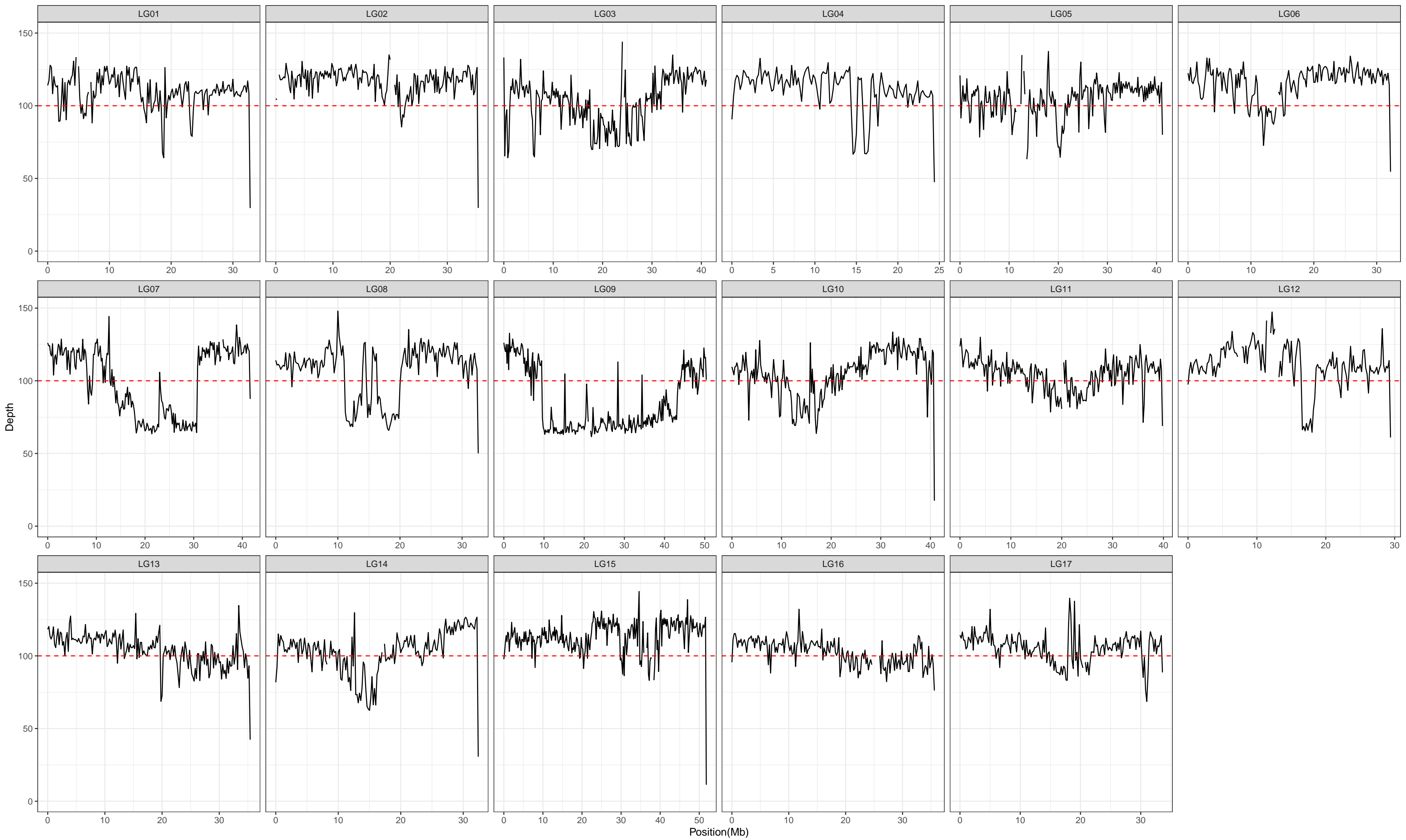

Supplement: Web_Material_uhad183 [file web_material_uhad183.zip › Fig. S15 The distribution depth map of the data on each chromosome of C. speciosa.pdf]

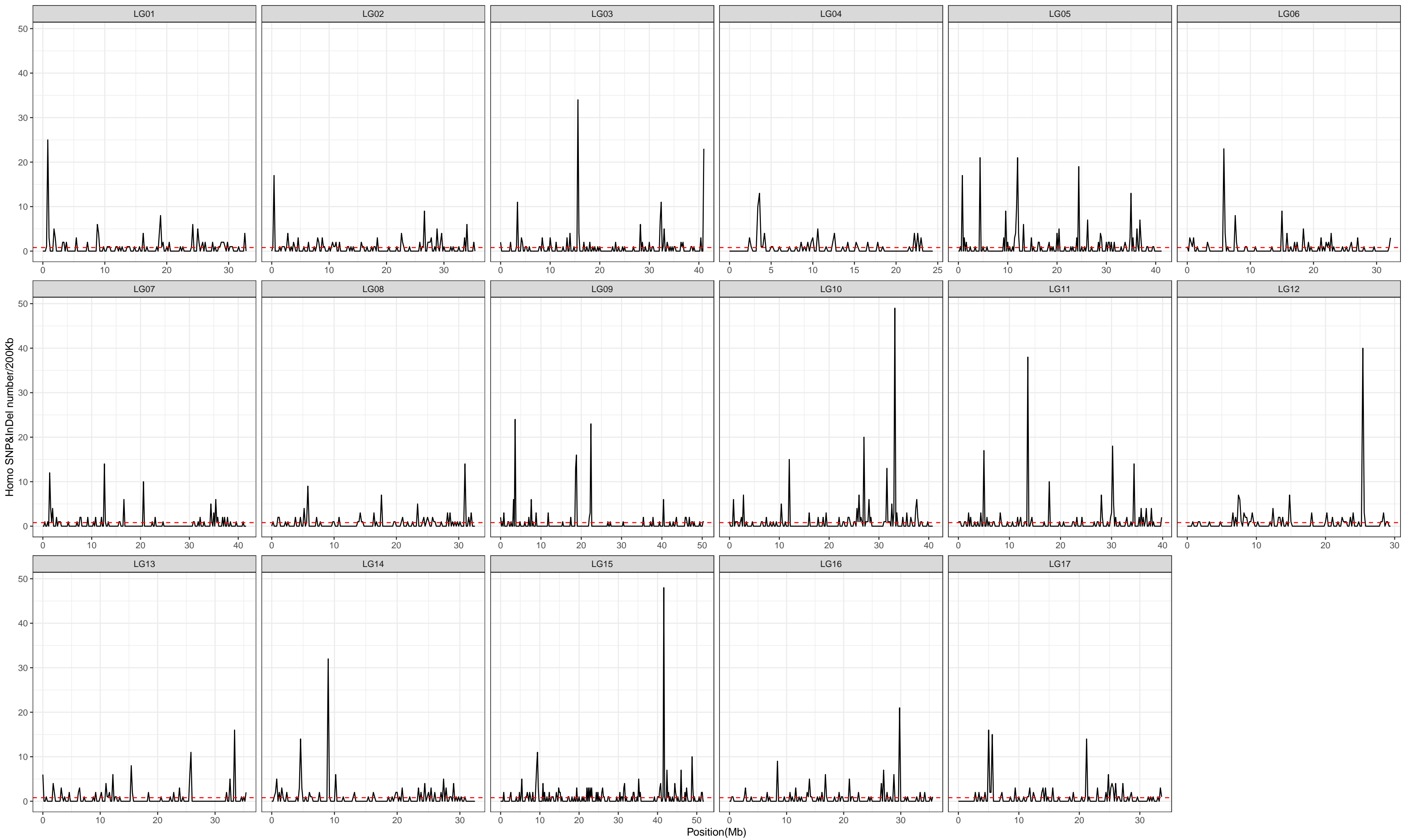

Supplement: Web_Material_uhad183 [file web_material_uhad183.zip › Fig. S16 The quantitative distribution of homozygous SNP_InDel on each chromosome of C. speciosa.pdf]

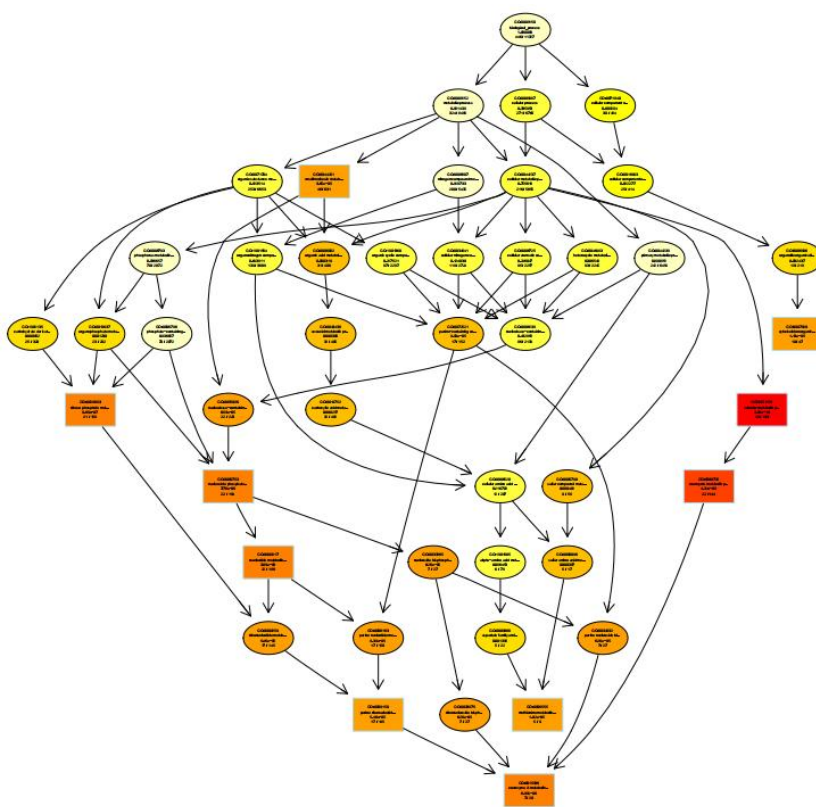

Biological process

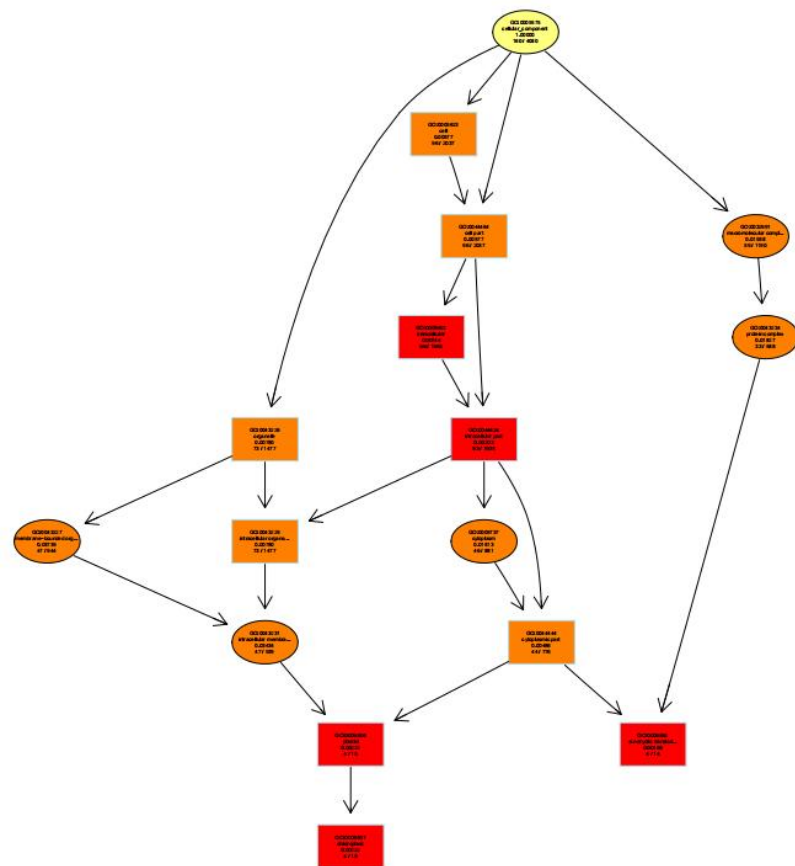

Cell component

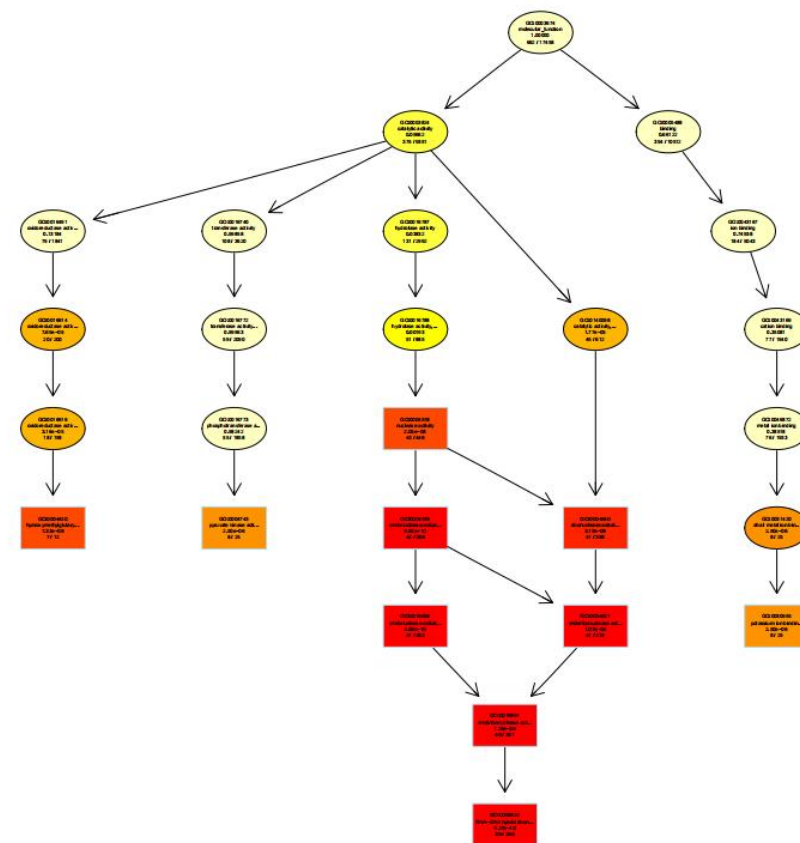

Molecular function

Supplement: Web_Material_uhad183 [file web_material_uhad183.zip › Fig. S17 GO enrichment analysis on the species-specific gene families of C. speciosa.pdf]

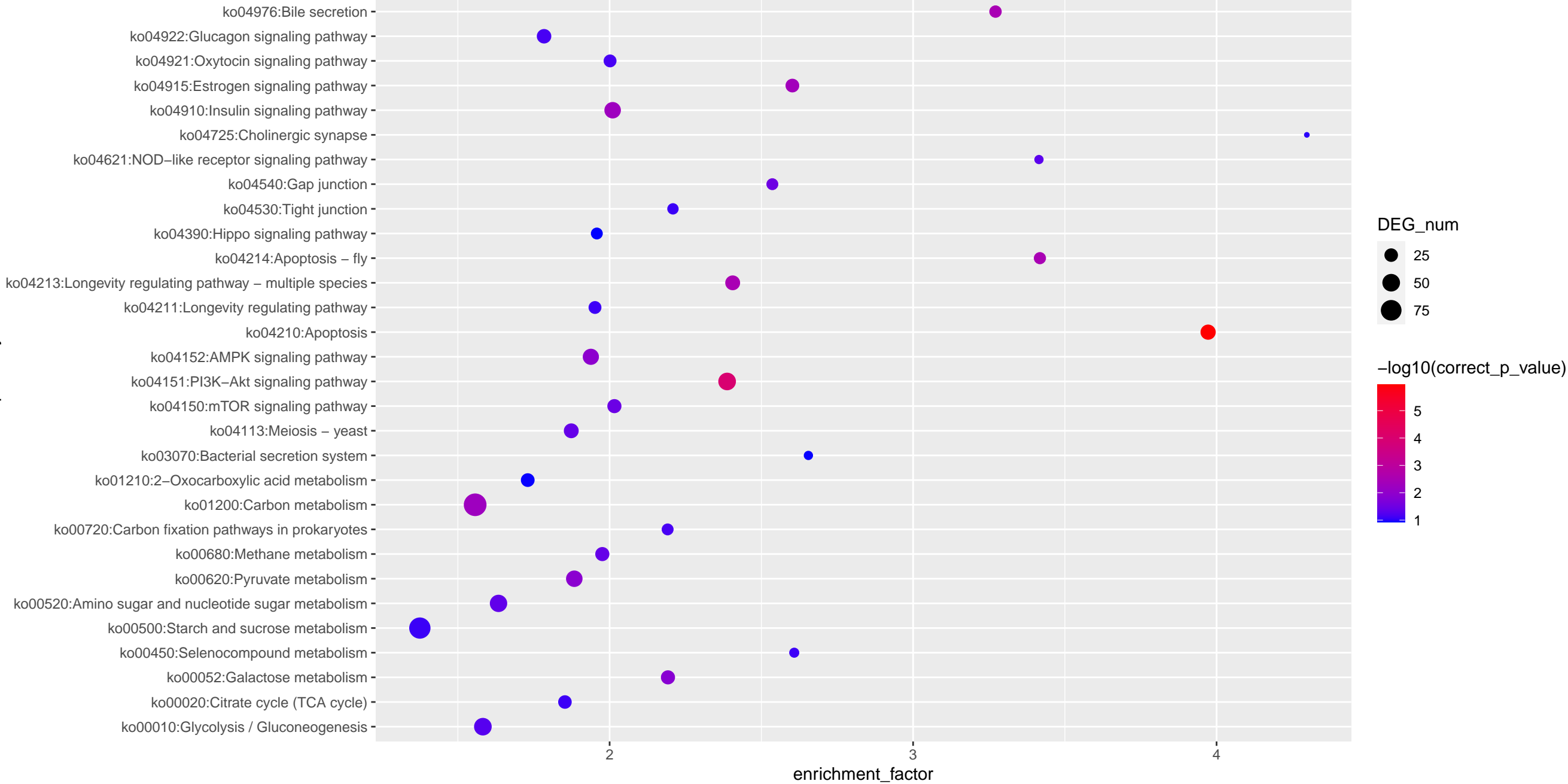

Supplement: Web_Material_uhad183 [file web_material_uhad183.zip › Fig. S18 KEGG enrichment analysis on the species-specific gene families of C. speciosa.pdf]

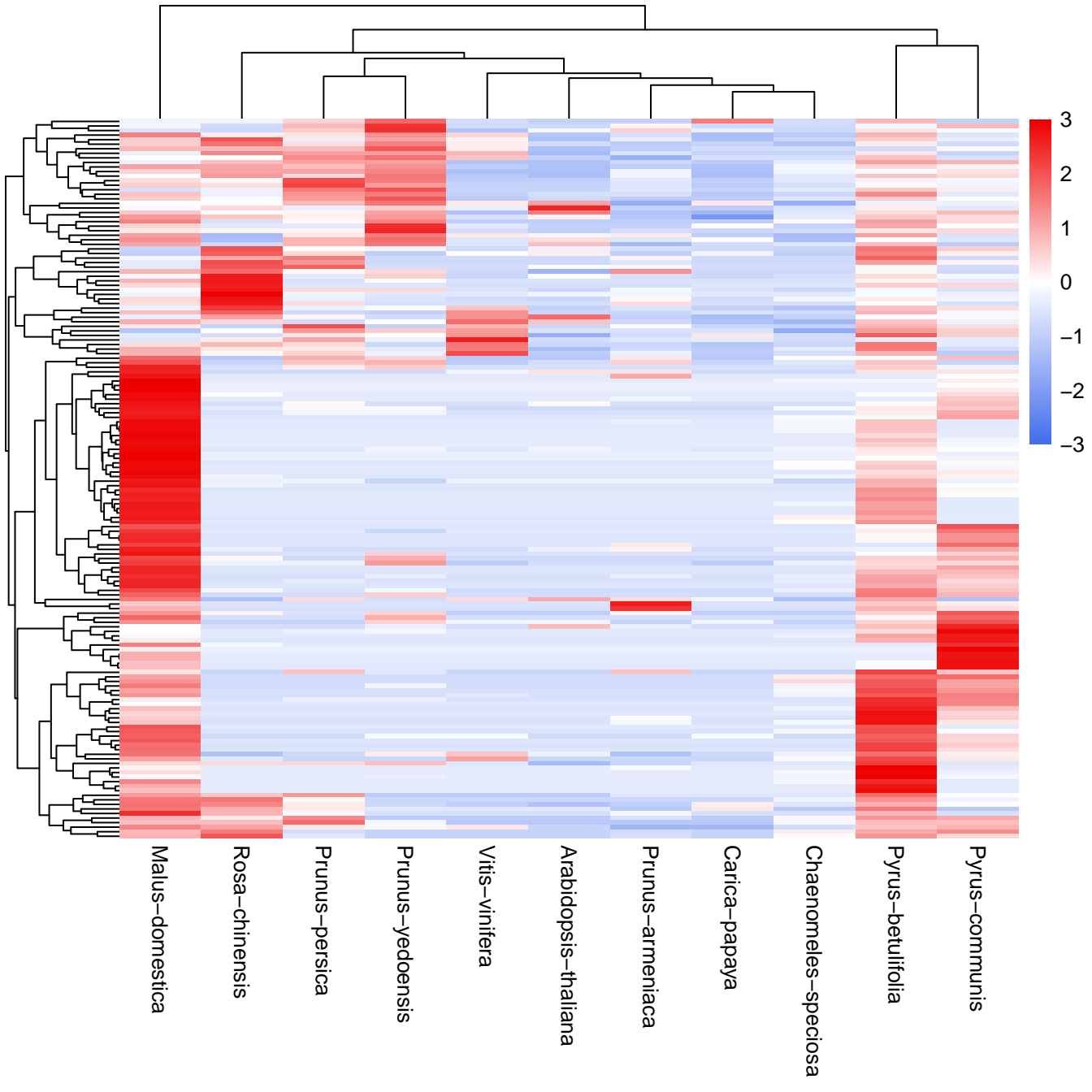

Supplement: Web_Material_uhad183 [file web_material_uhad183.zip › Fig. S19 Contraction family number heatmap and cluster analysis of C.speciosa..pdf]

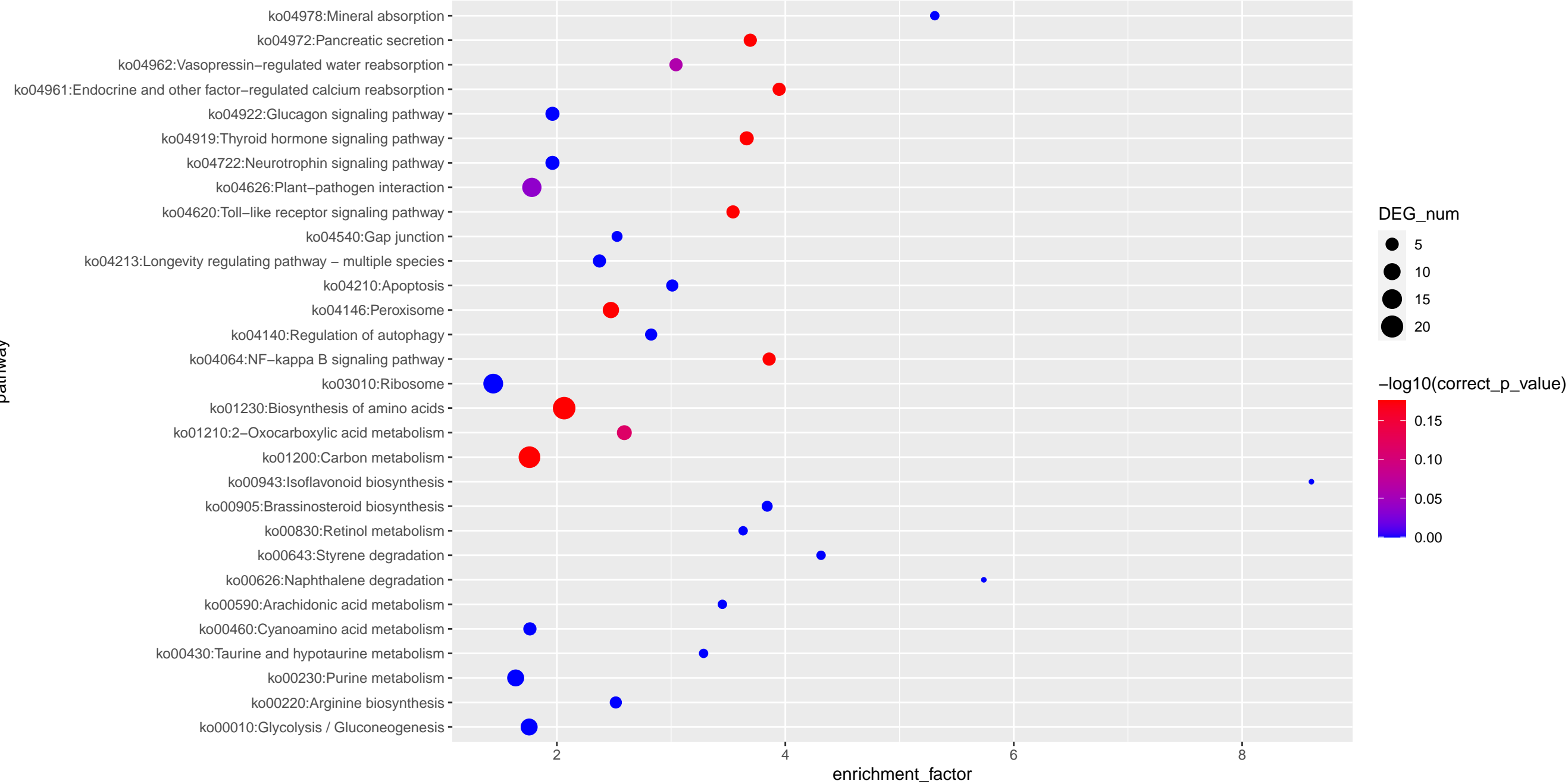

Supplement: Web_Material_uhad183 [file web_material_uhad183.zip › Fig. S2 kegg enrichment analysis on the species-specific gene families of Hap1.pdf]

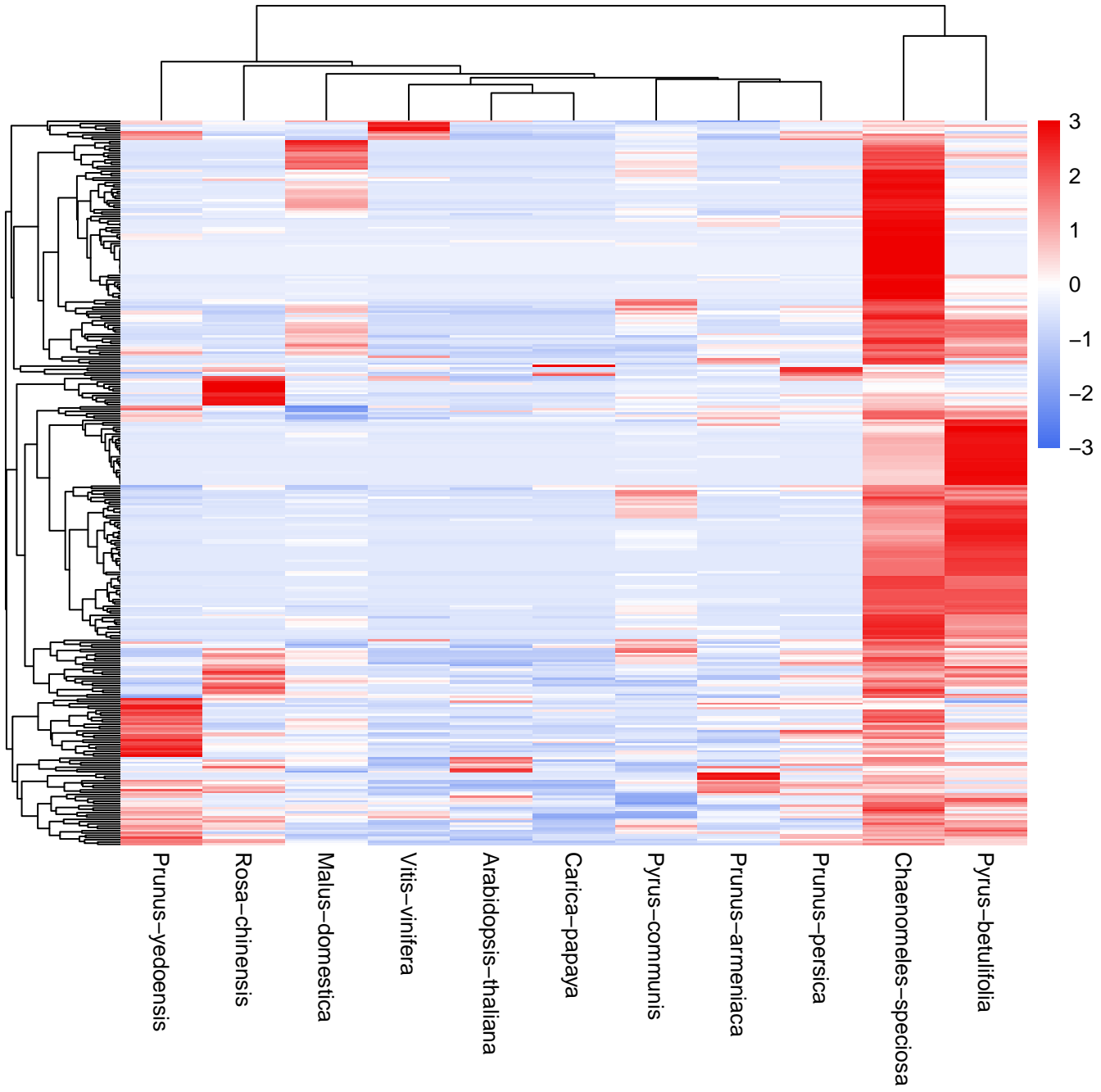

Supplement: Web_Material_uhad183 [file web_material_uhad183.zip › Fig. S20 Expansion family number heatmap and cluster analysis of C.speciosa.pdf]

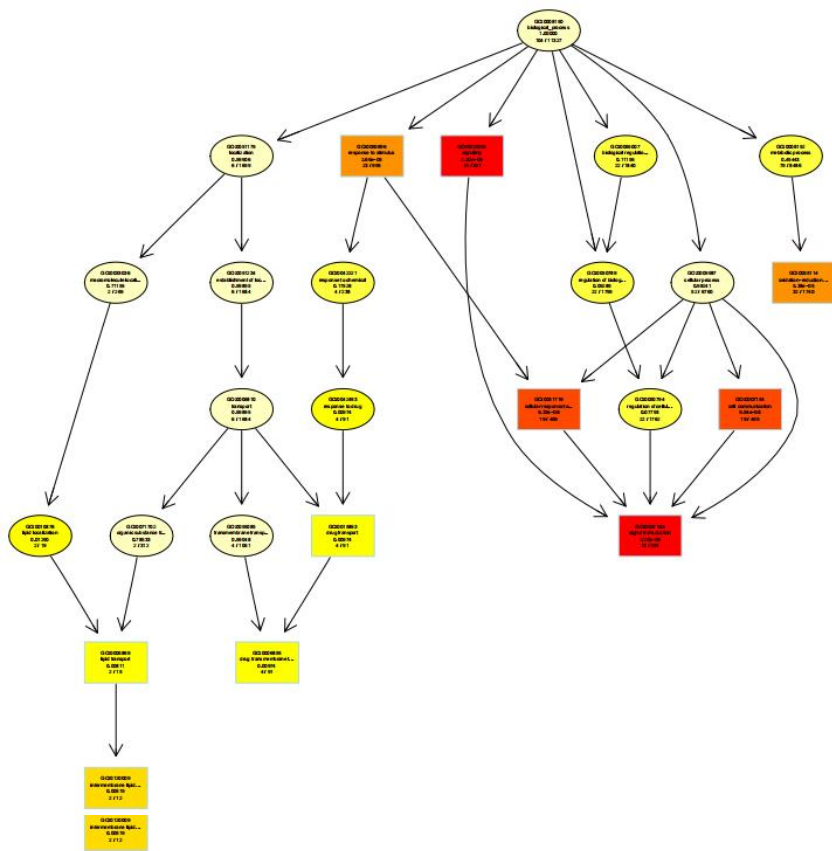

Biological process

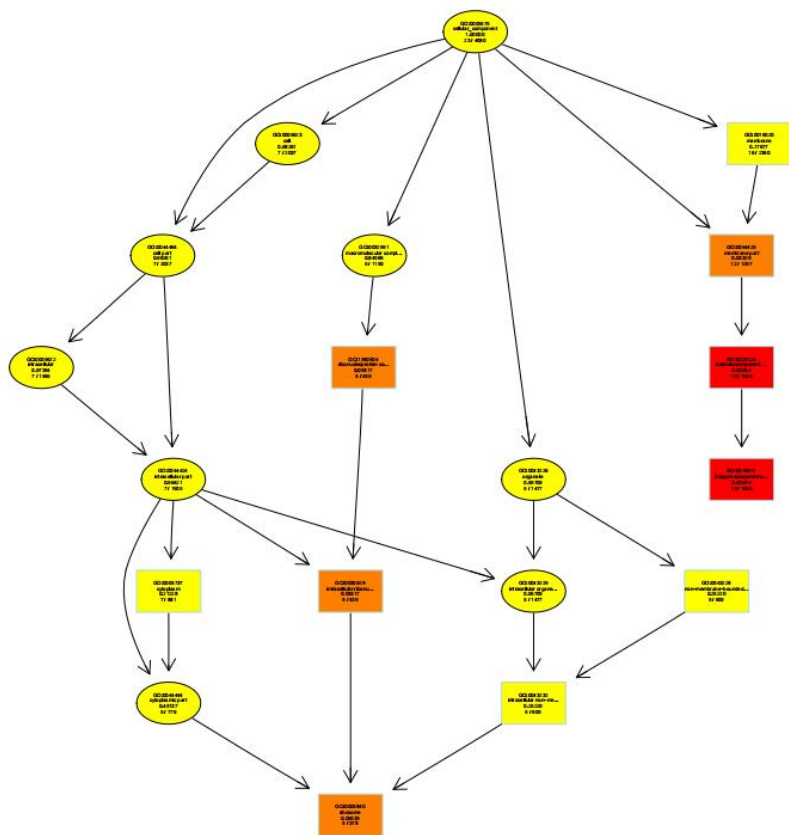

## Cell component

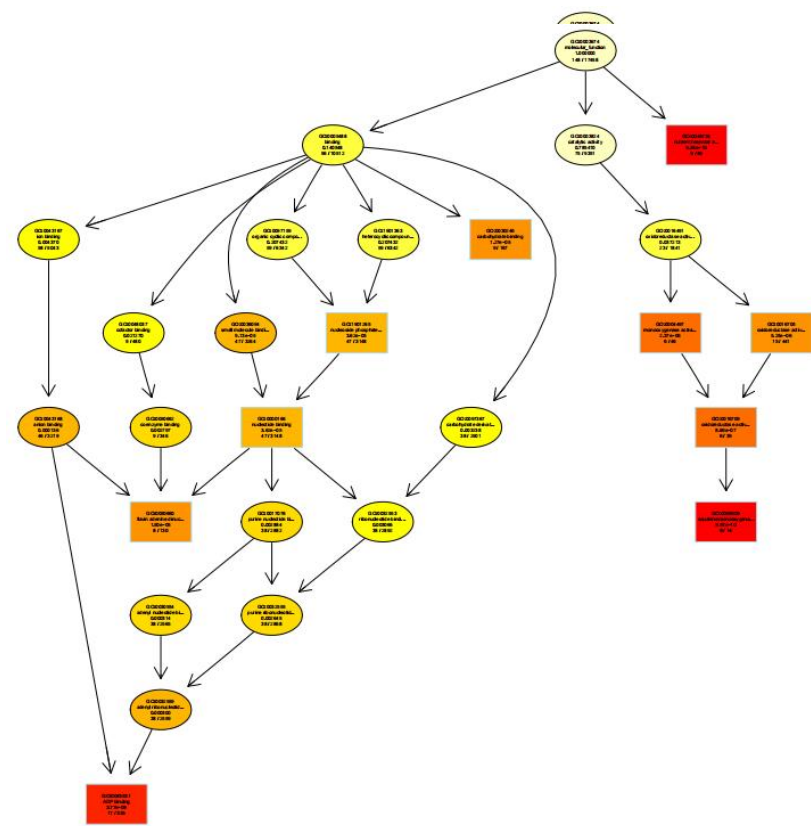

## Molecular function

Supplement: Web_Material_uhad183 [file web_material_uhad183.zip › Fig. S21 GO enrichment analysis on the contracted gene families of C. speciosa.pdf]

pathway

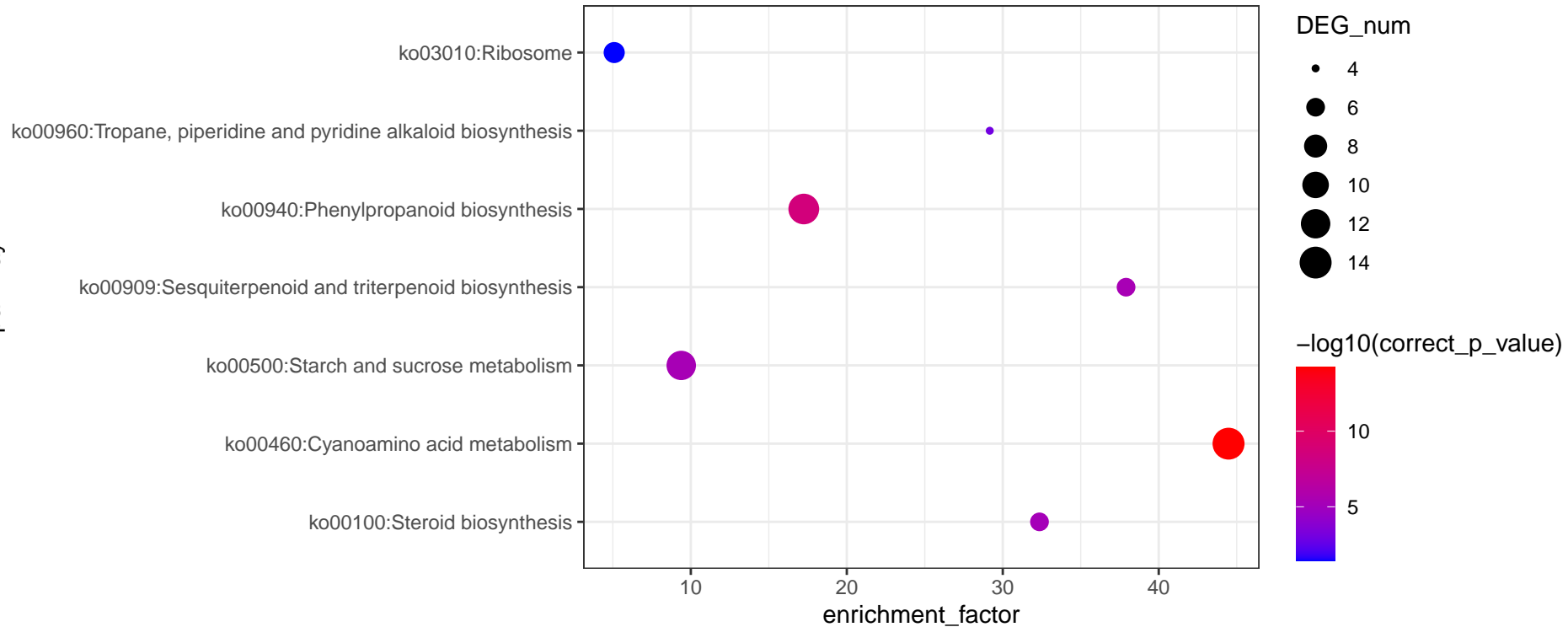

Supplement: Web_Material_uhad183 [file web_material_uhad183.zip › Fig. S22 KEGG enrichment analysis on the contracted gene families of C. speciosa.pdf]

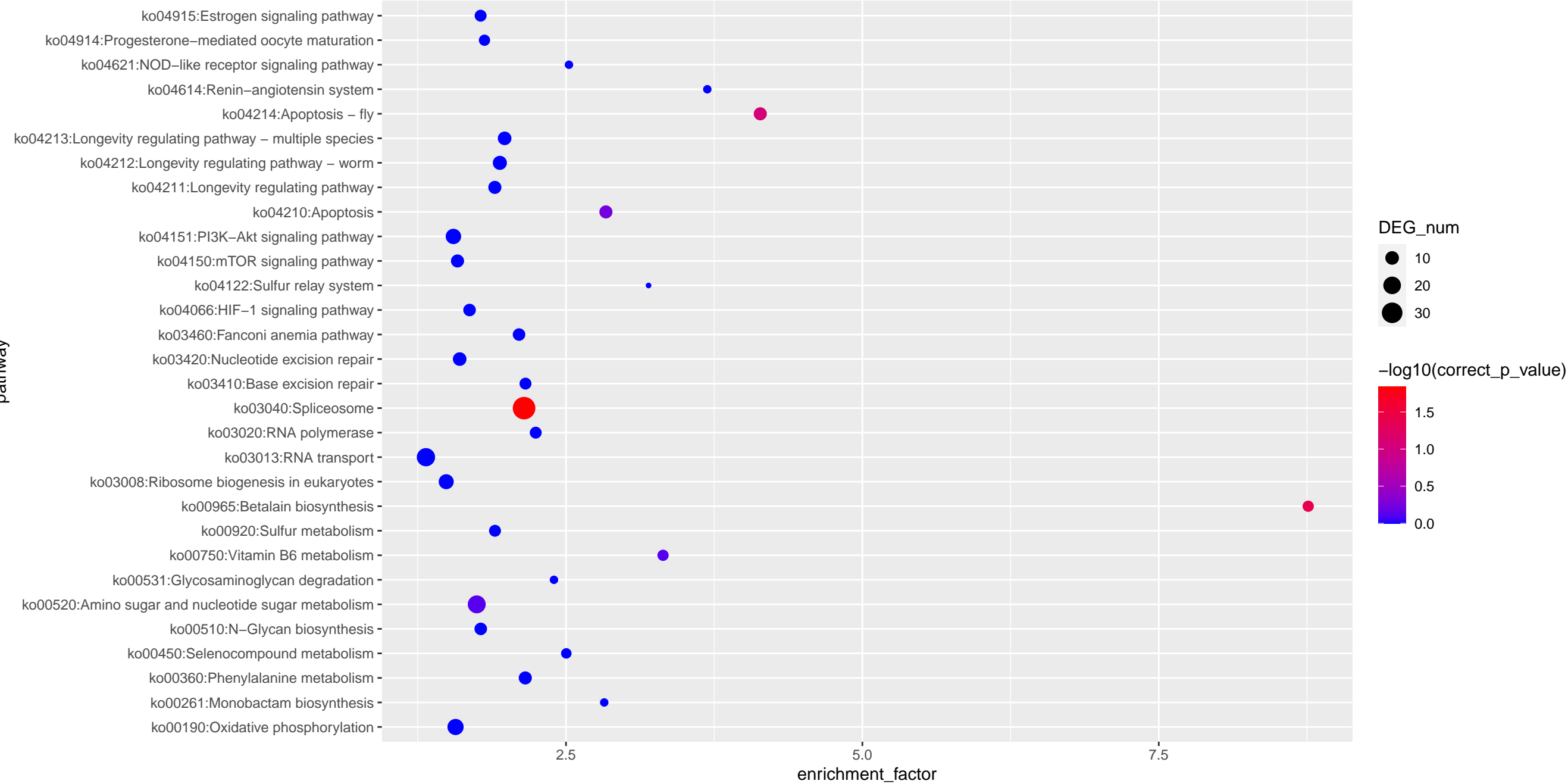

Supplement: Web_Material_uhad183 [file web_material_uhad183.zip › Fig. S3 kegg enrichment analysis on the species-specific gene families of Hap2.pdf]

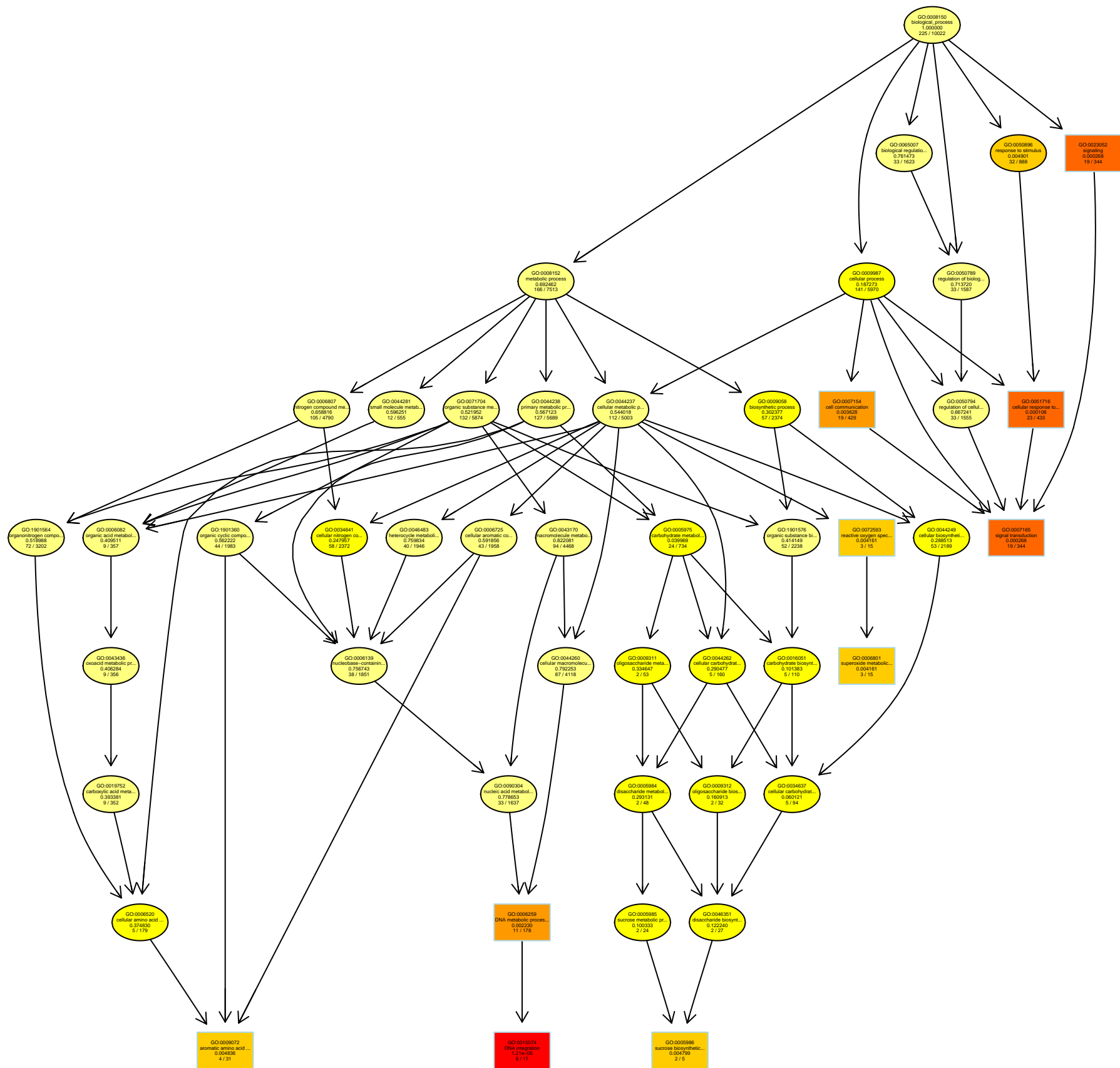

Supplement: Web_Material_uhad183 [file web_material_uhad183.zip › Fig. S4 GO enrichment analysis on the species-specific gene families of Hap1 BP_topGO_results.pdf]

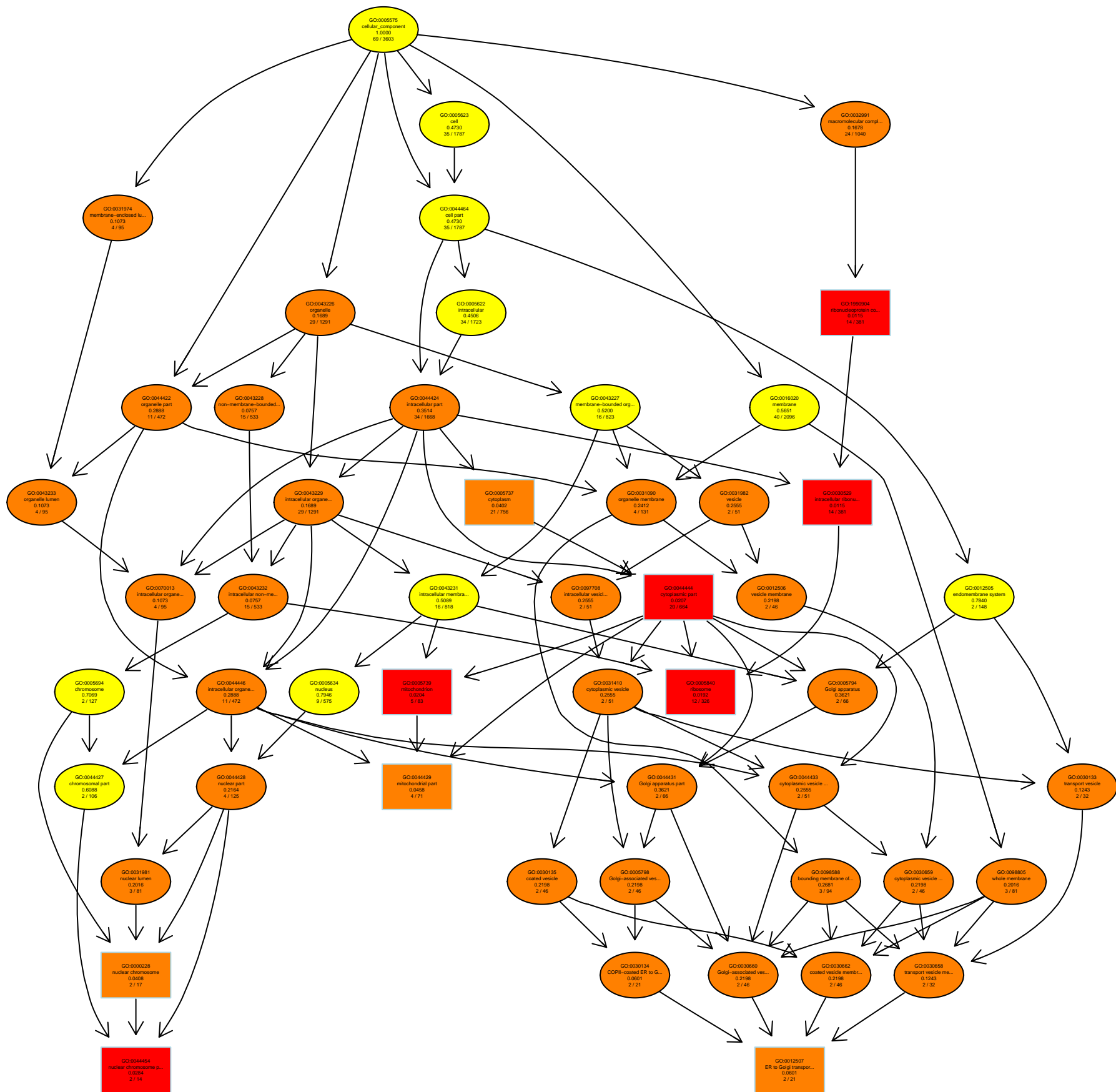

Supplement: Web_Material_uhad183 [file web_material_uhad183.zip › Fig. S5 GO enrichment analysis on the species-specific gene families of Hap1 CC_topGO_results.pdf]

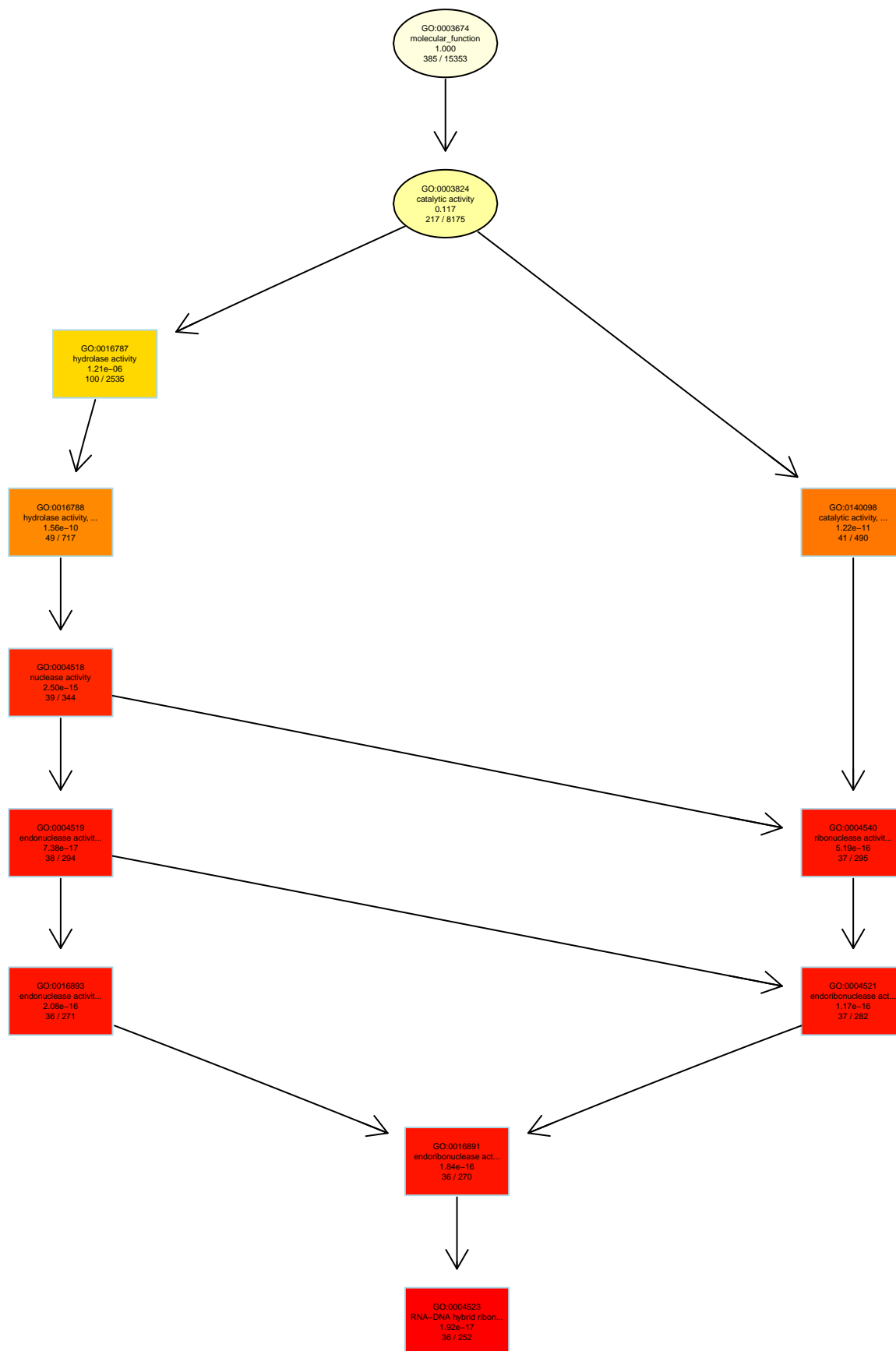

Supplement: Web_Material_uhad183 [file web_material_uhad183.zip › Fig. S6 GO enrichment analysis on the species-specific gene families of Hap1 MF_topGO_results.pdf]

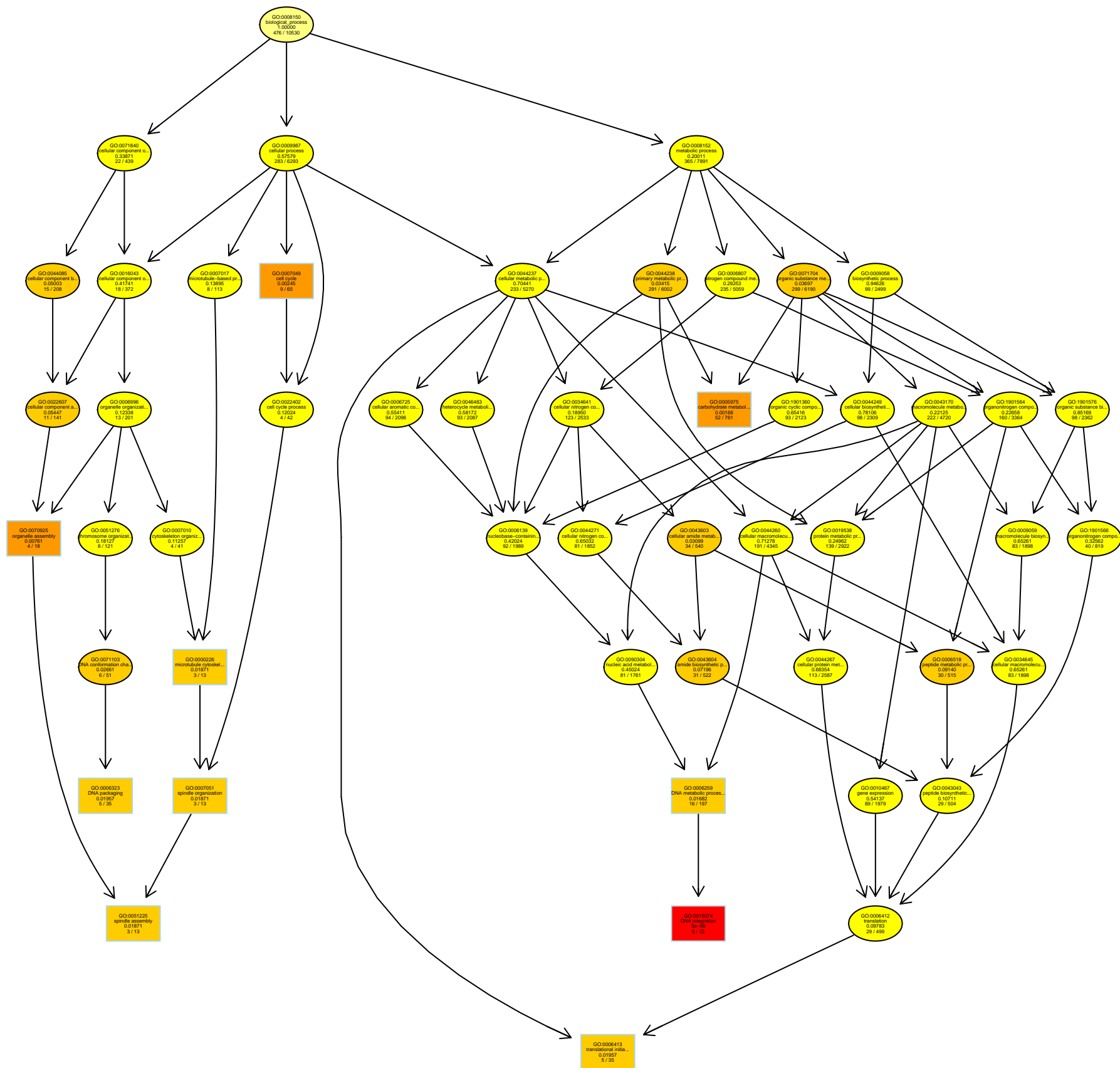

Supplement: Web_Material_uhad183 [file web_material_uhad183.zip › Fig. S7 GO enrichment analysis on the species-specific gene families of Hap2 BP_topGO_results.pdf]

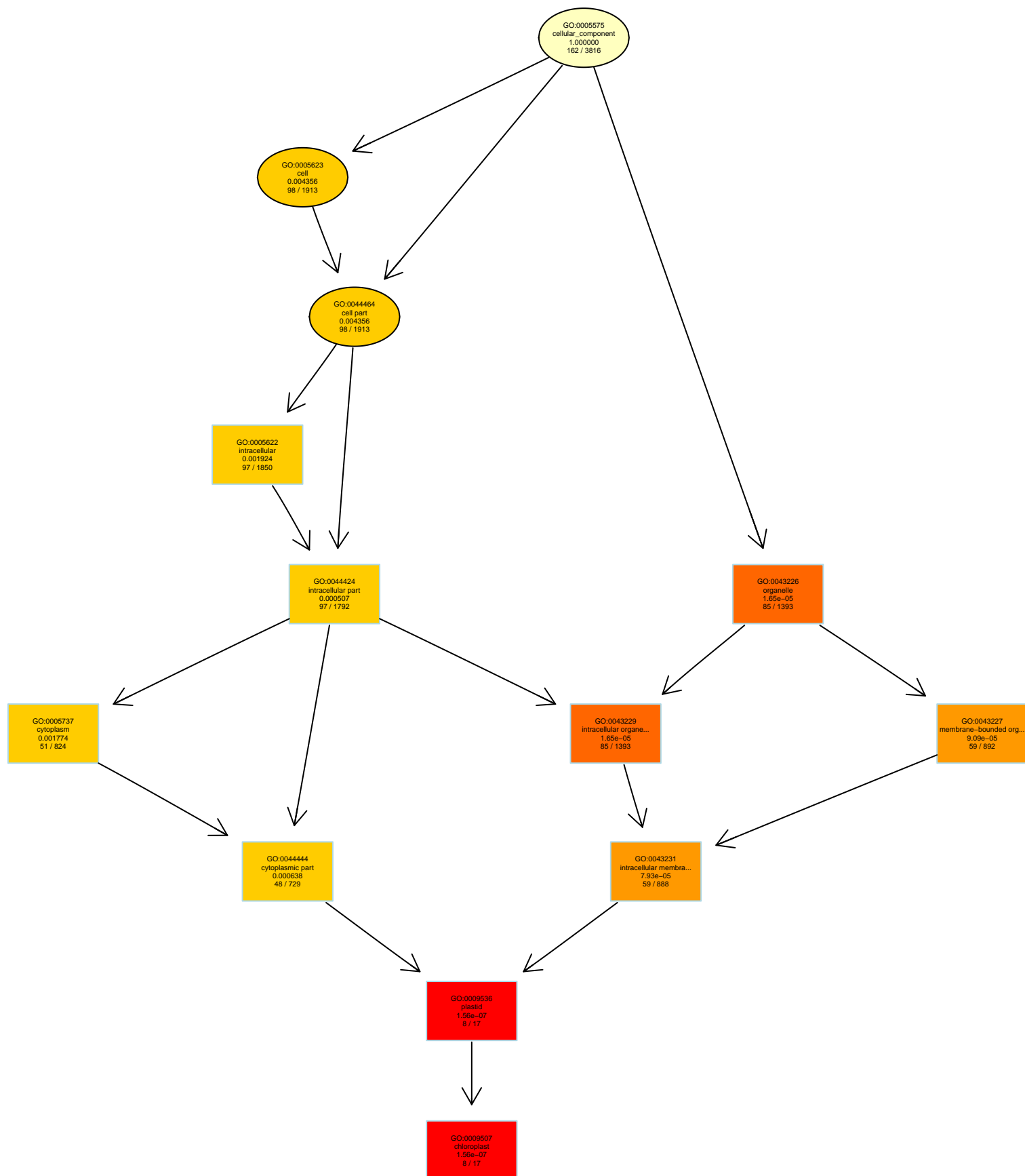

Supplement: Web_Material_uhad183 [file web_material_uhad183.zip › Fig. S8 GO enrichment analysis on the species-specific gene families of Hap2 CC_topGO_results.pdf]

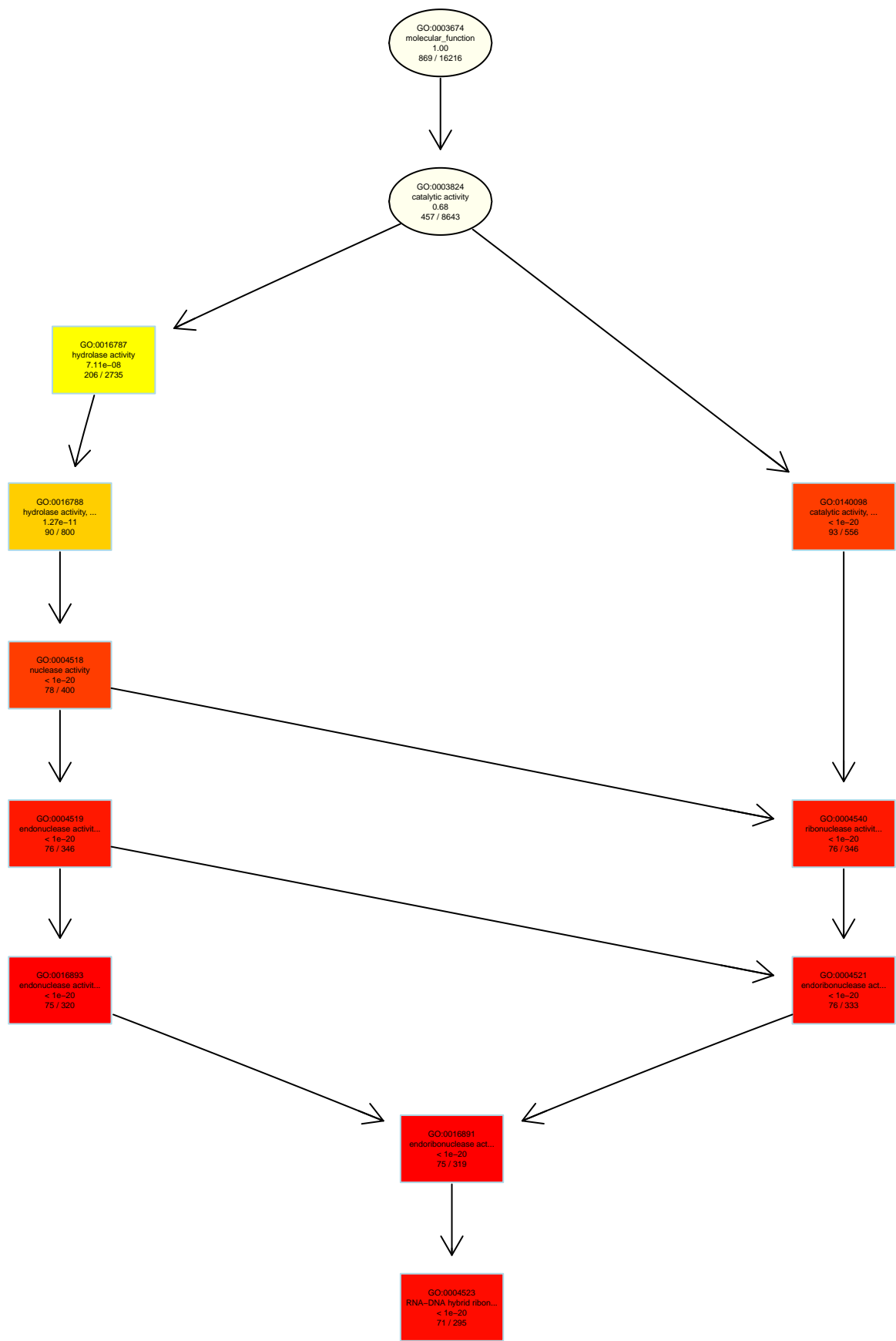

Supplement: Web_Material_uhad183 [file web_material_uhad183.zip › Fig. S9 GO enrichment analysis on the species-specific gene families of Hap2 MF_topGO_results.pdf]
