# Supplementary material for: A telomere-to-telomere reference genome provides genetic insight into the pentacyclic triterpenoid biosynthesis in Chaenomeles speciosa
Source: Hortic Res. 2023 Sep 14;10(10):uhad183. doi: 10.1093/hr/uhad183 (PMC10623406; doi:10.1093/hr/uhad183)
Supplement: Web_Material_uhad183 [file Fig. S13 The overall landscape of centromere motif density and inter-chromosomal Hi-C interaction strength in C. speciosa chromosomes.pdf › Fig. S13 The overall landscape of centromere motif density and inter-chromosomal Hi-C interaction strength in C. speciosa chromosomes.pdf]

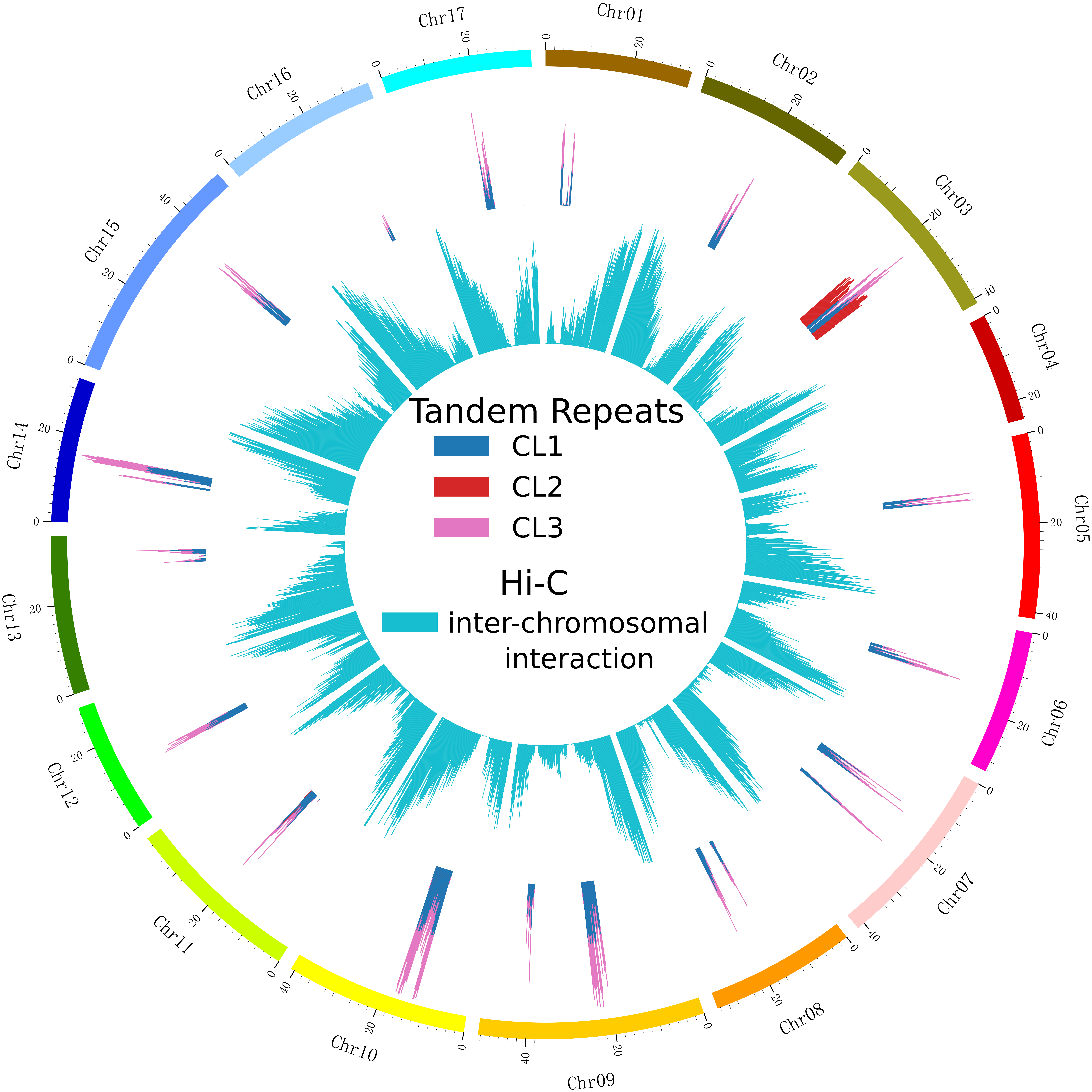

Tandem Repeats
CL1
CL2
CL3
Hi-C
inter-chromosomal interaction
Chr01
Chr02
Chr03
Chr04
Chr05
Chr06
Chr07
Chr08
Chr09
Chr10
Chr11
Chr12
Chr13
Chr14
Chr15
Chr16
Chr17
